# Gene Expression Analysis of Rice Seedling under Potassium Deprivation Reveals Major Changes in Metabolism and Signaling Components

**DOI:** 10.1371/journal.pone.0070321

**Published:** 2013-07-29

**Authors:** Alka Shankar, Amarjeet Singh, Poonam Kanwar, Ashish Kumar Srivastava, Amita Pandey, Penna Suprasanna, Sanjay Kapoor, Girdhar K. Pandey

**Affiliations:** 1 Department of Plant Molecular Biology, University of Delhi South Campus, Dhaula Kuan, New Delhi, India; 2 Nuclear Agriculture and Biotechnology Division, Bhabha Atomic Research Centre, Mumbai, India; Nanjing Agricultural University, China

## Abstract

Plant nutrition is one of the important areas for improving the yield and quality in crops as well as non-crop plants. Potassium is an essential plant nutrient and is required in abundance for their proper growth and development. Potassium deficiency directly affects the plant growth and hence crop yield and production. Recently, potassium-dependent transcriptomic analysis has been performed in the model plant *Arabidopsis*, however in cereals and crop plants; such a transcriptome analysis has not been undertaken till date. In rice, the molecular mechanism for the regulation of potassium starvation responses has not been investigated in detail. Here, we present a combined physiological and whole genome transcriptomic study of rice seedlings exposed to a brief period of potassium deficiency then replenished with potassium. Our results reveal that the expressions of a diverse set of genes annotated with many distinct functions were altered under potassium deprivation. Our findings highlight altered expression patterns of potassium-responsive genes majorly involved in metabolic processes, stress responses, signaling pathways, transcriptional regulation, and transport of multiple molecules including K^+^. Interestingly, several genes responsive to low-potassium conditions show a reversal in expression upon resupply of potassium. The results of this study indicate that potassium deprivation leads to activation of multiple genes and gene networks, which may be acting in concert to sense the external potassium and mediate uptake, distribution and ultimately adaptation to low potassium conditions. The interplay of both upregulated and downregulated genes globally in response to potassium deprivation determines how plants cope with the stress of nutrient deficiency at different physiological as well as developmental stages of plants.

## Introduction

Potassium is one of the essential macronutrients required for plant growth and development. It plays a major role in different physiological processes like cell elongation, stomatal movement, turgor regulation, osmotic adjustment, and signal transduction by acting as a major osmolyte and component of the ionic environment in the cytosol and subcellular organelles [1]–[7]. Potassium is also required for balancing the electrical charge of membranes, energy generation by proton pump activity, long-distant transport of ions from root to shoot, protein synthesis, enzyme activation, and metabolism of sugars and nitrogen [2], [8], [9]. Since potassium is one of the major plant macronutrients (cytosolic K^+^ concentration is approximately 100 mM), potassium deficiency poses a severe agricultural challenge and requires the use of large quantities of chemical fertilizers for sustainable agricultural practices.

Previous studies report that potassium acts as an activator or cofactor in several enzyme systems [10]. Enzymatic activity of pyruvate kinase, starch synthase, nitrate reductase, and rubisco are all directly connected to metabolic changes under potassium deficiency [11]–[14]. One of the hallmarks of potassium deficiency is chlorosis (yellowing) in older leaves, a consequence of mobilization of potassium from older leaves to younger growing tissues [2], [9], [15].

Potassium uptake takes place in the roots of plants; potassium is subsequently redistributed to plant tissues and organs and stored in abundance in vacuoles. Plant roots tolerate short-term potassium deprivation by utilizing potassium stored in the vacuole when available. When plants grow in potassium-deficient soil, the root cells sense the low concentrations of K^+^ and initiate a series of physiological reactions [16], [17]. The detailed physiological role of potassium absorption and uptake has been studied in several plant species, and the molecular mechanisms of potassium transport have been largely elucidated in *Arabidopsis*. A large number of transporters and channels in *Arabidopsis* have been implicated in the uptake and mobilization of potassium from root to other parts of the plant [4], [18]–[21]. To adjust fluctuation of potassium levels in the soil, plants have adopted two modes of potassium uptake in their roots, namely high-affinity and low-affinity uptake [4], [22], [23]. Recently, studies implicated calcium-mediated CBL-CIPK signaling in regulating the shaker family K^+^ channels AKT1 and AKT2, however detailed mechanistics of potassium sensing remain elusive [6], [24], [25]. Research related to the molecular mechanisms of K^+^ sensing, uptake, distribution, and homeostasis in cereal and non-cereal crops is still miniscule. Although considerable work has been done in the model plant *Arabidopsis*, an extensive amount of work is still needed in crop plants to understand the detail mechanisms of K^+^ nutrition and signaling.

In this study, we used whole genome microarrays to determine the transcriptomic profile of rice seedlings exposed to short-term K^+^ deficiency followed by K^+^ resupply. We applied Benjamini-Hochberg correction to filter the differentially expressed genes in different conditions. We also performed PCA (Principal Component Analysis) and Pearson correlation coefficient analysis to ensure reliability of the data. According to our microarray data, potassium deficiency affects the expression of various genes, which were grouped into different categories such as metabolism, transcription factor, transporter, signal transduction. The objective of this study is to determine the expression pattern of genes under low potassium stress conditions and to determine the role of these genes in potassium homeostasis and adaptation in potassium-deficient soil.

## Results

### Phenotypic Analysis of K^+^ Deficiency on Rice Seedling Growth

**Figure 1:**
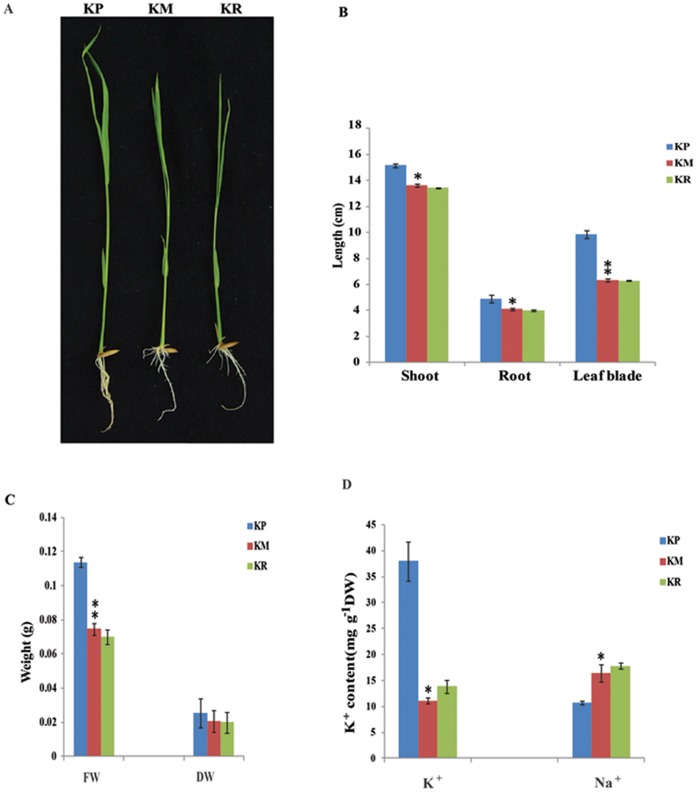
Growth and ion content analysis of rice seedlings during potassium nutrient deficiency. (**A**) Analysis was performed 5 days after transfer of seedling in KP and KM medium. Phenotypes of rice seedlings under normal, K^+^ deficient and resupply conditions (KP, KM and KR). (**B**) Length of rice shoot, root and leaf blade under three different conditions. (**C**) Fresh and dry weight comparison of rice seedling. (**D**) K^+^ and Na^+^ content of rice seedlings. Differences between mean values of treatments and controls were compared using t - tests (* P<0.05, ** P<0.01).

For transcriptional analysis under potassium deficiency, 5 days old hydroponically grown indica rice IR64 seedlings were transferred to a potassium-deficient medium for 5 days followed by resupply of potassium for 6 h. Seedlings grown in potassium-deficient media had reduced shoot and root growth and leaf blade height (Figure 1A, B). However, there was no phenotypic difference between K^+^ minus or starved (KM) and K^+^ resupply (KR) seedlings after treatment for 6 h. In addition, we observed a higher number of root hairs in KM seedlings while more lateral roots were seen in K^+^ plus or normal growth (KP) seedlings (Figure 1A, B). The biomass and ion content (K^+^, Na^+^) of seedlings were also measured for each condition. The fresh and dry weight of KM seedlings was lower after 5 days of K^+^ deficiency compared to normal growth conditions, whereas no significant difference was observed between KM and KR (Figure 1C). In order to determine the total K^+^ and Na^+^ content, single-channel flame photometry (see Material and Methods) was performed for all the three conditions. KM seedlings contained about 4 times less K^+^ than KP seedlings while KR showed slightly higher K^+^ than KM plants (Figure 1D). Na^+^ content was lower in KP condition as compared to KM and KR condition, however no significant difference was found in KM and KR seedlings.

### Global Expression Analysis in Response to Potassium Deficiency and Resupply Condition

**Figure 2:**
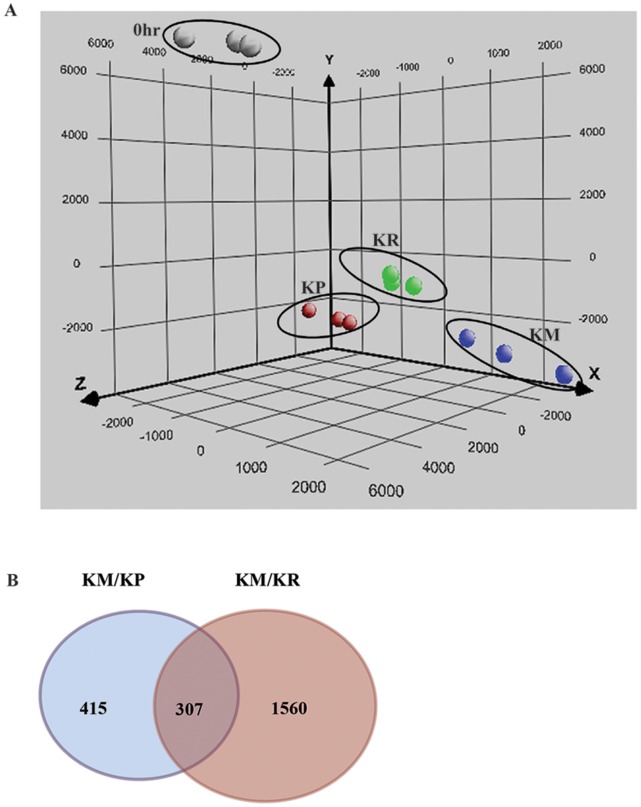
Overview of the changes in transcripts in potassium sufficient, deficient and resupply condition. (**A**) Principal component analysis (PCA) of the changes in transcript abundance in rice seedling under different condition. (**B**) Venn diagram showing the number of differentially expressed genes (P<0.05 and fold change equal or more than 2) in response to KM/KP and KR/KM in seedling.

To obtain insights into changes in rice gene expression profiles under potassium deprivation and resupply of potassium, we performed rice whole genome microarrays using Affymetrix gene chip (57 K). Rice seedlings were subjected to three different conditions, viz. K^+^ plus (KP), K^+^ minus (KM), and K^+^ resupply (KR) after five days of normal growth as described in the material and method. Total RNA was isolated from treated seedlings and used for whole genome microarray analysis. The microarray gene expression data was normalized against data obtained for samples grown on normal media for five days (untreated seedlings) in order to eliminate potassium-responsive genes involved in early growth and development of seedlings. To determine the accuracy and reliability of the data obtained, microarray data of three biological replicates for each treatment was analysed for their correlation coefficient, and Principal Component Analysis (PCA) was also performed. Correlation between the biological replicates was calculated using Pearson’s correlation coefficient based on the signal intensities. The three replicates of each treatment showed correlation coefficient of greater than 0.98 (Table 1). The values of the correlation coefficients of the three replicates were further reinforced by the result of the PCA, wherein the biological replicates for each treatment showed a high degree of correlation. Interestingly, the PCA cluster for the third treatment i.e. resupply of K^+^-deficient medium with K^+^ for 6 h (KR) was positioned closer to the PCA cluster of the K^+^-plus condition (KP) as compared to the PCA cluster for the K^+^-minus condition (KM) (Figure 2A). This indicated that resupplying potassium externally for 6 h restored expression of many genes to their normal levels [i.e. K^+^ plus condition (KP)].

**Table 1:** Correlation data from microarray analysis.

Array Name	KR1	KR2	KR3	0 hr1	0 hr2	0 hr3	KP1	KP2	KP3	KM1	KM2	KM3
**KR1**	1	0.9942733	0.991799	0.8602868	0.8646402	0.8550841	0.945861	0.958903	0.960618	0.940772	0.949816	0.948117
**KR2**	0.9942733	1	0.9917473	0.8624464	0.8658453	0.8552977	0.944316	0.959933	0.960965	0.938354	0.947319	0.945787
**KR3**	0.991799	0.9917473	1	0.8662803	0.8702267	0.8627964	0.944466	0.954373	0.958572	0.935967	0.942745	0.940963
**0 hr1**	0.8602868	0.8624464	0.8662803	1	0.9964587	0.9892928	0.875728	0.879222	0.874045	0.869435	0.857152	0.858365
**0 hr2**	0.8646402	0.8658453	0.8702267	0.9964587	1	0.9915484	0.873703	0.878731	0.874345	0.869253	0.858916	0.859034
**0 hr3**	0.8550841	0.8552977	0.8627964	0.9892928	0.9915484	1	0.863212	0.864016	0.859468	0.861733	0.850758	0.85059
**KP1**	0.945861	0.9443161	0.9444658	0.875728	0.8737032	0.8632119	1	0.989603	0.984926	0.946355	0.944624	0.946672
**KP2**	0.9589027	0.9599334	0.9543728	0.8792217	0.8787307	0.8640156	0.989603	1	0.992018	0.950186	0.953336	0.954741
**KP3**	0.9606177	0.9609647	0.9585716	0.8740448	0.8743453	0.8594683	0.984926	0.992018	1	0.952037	0.956309	0.957403
**KM1**	0.9407717	0.938354	0.9359667	0.8694348	0.8692533	0.8617328	0.946355	0.950186	0.952037	1	0.992166	0.992698
**KM2**	0.9498156	0.9473191	0.9427447	0.8571518	0.8589162	0.8507582	0.944624	0.953336	0.956309	0.992166	1	0.997317
**KM**	0.948117	0.9457873	0.9409633	0.8583649	0.8590338	0.8505895	0.946672	0.954741	0.957403	0.992698	0.997317	1

**Figure 3:**
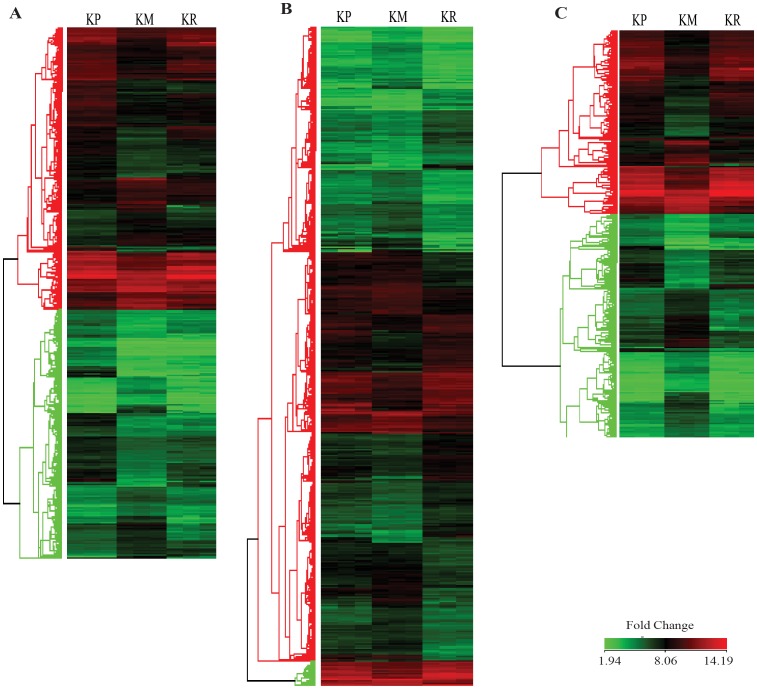
Hierarchical cluster analysis. (**A**) Hierarchical cluster analysis of the 722 potassium responsive genes found in potassium deficient condition, (**B**) 1876 genes found in potassium resupply condition and (**C**) 307 genes found common between potassium deficient and resupply condition, which were truly differentially expressing under K^+^ deficient conditions based on their reversal of expression upon resupply condition.

After normalization of the microarray data, differential expression analysis was performed for the following pairwise combinations of the three test conditions: KP and KM (KP/KM) and KM and KR (KM/KR). Differentially expressed genes were identified on the basis of following two criteria: first, the fold change, wherein genes with a fold change of ≥2 were considered for analysis and second, the unpaired t-test, wherein genes with p-value ≤0.05 were selected for analysis. False discovery rate (FDR) correction (Benjamini-Hochberg Correction) was applied, which added greater significance to the data. 722 genes were differentially expressed under potassium deficient (KM) condition while 1867 genes were differentially expressed upon potassium resupply (KR). The details (probe set Ids, signal intensity value, p-value, fold change and regulation) of 722 and 1867 genes is given in Table S1 and Table S2 respectively. Among these 722 differentially expressed genes, 281 genes showed up regulation and 441 genes were down regulated (Figure 3A). Out of the 1867 genes differentially expressed upon potassium resupply, 866 showed up regulation, while 1001 genes were down regulated (Figure 3B).

**Figure 4:**
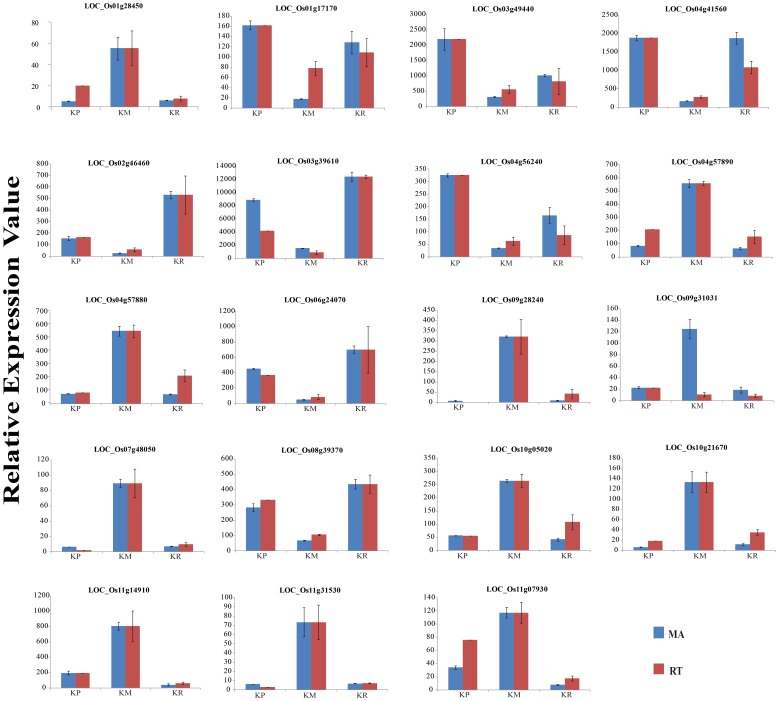
Confirmation of microarray data for selected genes by qRT-PCR analysis. Nineteen genes were selected and their expression profiles were assessed by qRT-PCR in all three conditions to verify the microarray data by using three biological replicates. Y-axis represents relative expression values obtained after normalizing the data against maximum expression value and X-axis shows different nutritional treatments. Blue bars represent the expression from microarrays, while red bars represent the qRT- PCR values.

A total of 307 differentially expressed genes were common for both KP and KR condition (Figure 2B, Figure 3C), details of which are given in Table S3. For the validation of microarray data, 19 significantly and differentially expressed genes representing different categories were selected for quantitative realtime PCR (qPCR) analysis. The results of qPCR of the 19 tested genes were in accordance with the microarray results, which authenticated our microarray data and established its reliability (Figure 4, Table S4). The raw data sets (CEL) and the normalized expression data sets have been deposited in the Gene Expression Omnibus at the National Center for Biotechnology Information with the accession number GSE44250 (http://www.ncbi.nlm.nih.gov/geo/).

### Functional Classification of the Differentially Expressed Genes

**Figure 5:**
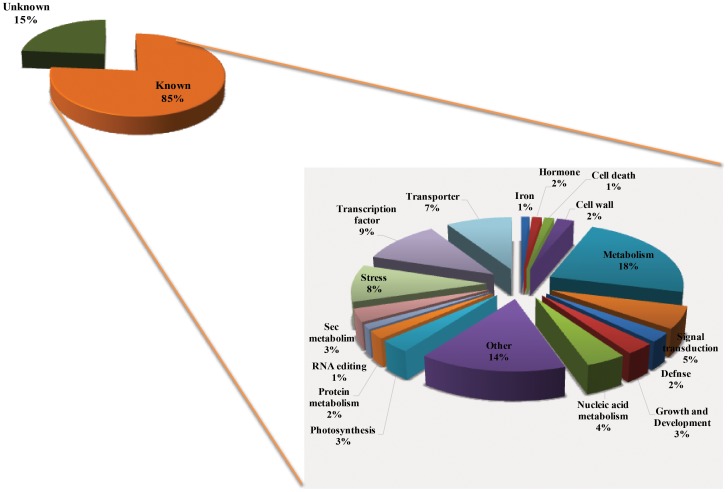
Functional categorization of potassium responsive genes into different categories. Detailed classification of genes showing transcriptional changes in the several categories based on the gene ontology (GO) and their putative function.

The differentially expressed genes in potassium-deficient conditions were functionally classified by homology search against the Gene Ontology (GO) and NCBI Non-redundant (NR) databases using BLAST through NCBI. Genes encoding hypothetical proteins were classified as genes of unknown function. Among the 722 genes that were differentially expressed under potassium deprivation, 15% of the genes belonged to the unknown function category (Figure 5). The remaining 85% genes were categorised into 17 comprehensive subdivisions corresponding to the following functions: primary metabolism, secondary metabolism, nucleic acid metabolism, transporters, transcription factors, auxin signaling components, cell wall metabolism, cell death, growth and development, photosynthesis, stress responses, etc. (Figure 5). Genes that could not be classified into any of the above-mentioned categories were placed in the category of “others”. Overall, this analysis indicate that potassium deficiency affects the expression of genes with diverse functions in metabolism, transcriptional regulation, stress responses, molecular transport and signal transduction. In all, nearly 56% of total differentially expressed genes are involved in these processes according to their annotation.

18% of differentially expressing genes (DEG) were involved in various metabolic pathways, including 4% in nucleic acid metabolism, 3% in secondary metabolism and 2% in protein metabolism, carbohydrate metabolism (primary metabolism), lipid metabolism, glycolytic enzymes and other biosynthetic pathways (Table 2). Of all the DEG, 7% were transporters, including three potassium transporters HAK1, *OsHKT2;3* and *OsHKT2;4*; ABC transporter; 3 peptide transporter PTR2 and PTR3-A, and many other diverse transporters. Diverse group of kinases (CIPKs and MAPKs), phosphatases (PP2C and other Ser/Thr protein phosphatases), and calcium sensors (CMLs and CBLs) known to be involved in signal transduction mechanism constituted 5% of DEG. Significant percentages of the total DEG were comprised of genes annotated as transcription factors (9%) and stress responsive genes (8%). A small group of genes related to photosynthesis (3%), growth and development (3%), cell wall (2%), defence (2%), and auxin (1%) were also differentially expressed under potassium-deficient conditions.

**Table 2:** Different category of differentially regulated genes in potassium deficient condition.

Probe Set ID	LOC_ID	Putative Function	FC in KM	Regulation in KM	FC in KR	Regulation in KR
**Carbohydrate metabolism**						
Os.9996.1.S1_at	LOC_Os11g10980	pyruvate kinase, putative, expressed	5.8973556	down	2.7624514	up
Os.57437.1.S1_at	LOC_Os02g52700	alpha-amylase precursor	16.978466	up	13.761532	down
Os.38240.1.S1_a_at	LOC_Os12g08760	carboxyvinyl-carboxyphosphonate phosphorylmutase, putative, expressed	62.562016	down	3.4629653	up
Os.33211.1.S1_at	LOC_Os09g28430	alpha-amylase precursor	49.027573	up	44.134583	down
Os.50903.1.S1_at	LOC_Os09g08130	indole-3-glycerol phosphate synthase, chloroplast precursor, putative, expressed	4.5081425	up	6.329595	down
Os.9212.1.S1_at	LOC_Os07g22930	starch synthase, putative, expressed	10.27882	down	1.8906631	up
Os.17814.2.S1_x_at	LOC_Os07g43700	lactate/malate dehydrogenase, putative, expressed	4.746752	down	4.3317947	up
**Lipid metabolism**						
Os.17491.1.S1_at	LOC_Os10g35070	Alpha-galactosidase precursor, putative, expressed	4.75564	down	2.4528239	up
Os.10339.1.S1_at	LOC_Os03g04770	beta-amylase, putative, expressed	8.125269	down	1.3109915	up
Os.46633.1.A1_at	LOC_Os10g38940	fatty acid hydroxylase, putative, expressed	3.8152332	down	2.6639307	up
Os.50047.1.S1_at	LOC_Os02g11680	glucosyltransferase, putative, expressed	3.1383889	down	1.010676	up
Os.7665.1.S1_at	LOC_Os04g56240	lipase, putative, expressed	9.613265	down	4.904837	up
Os.7985.1.S1_at	LOC_Os03g18070	omega-3 fatty acid desaturase, chloroplast precursor, putative, expressed	8.088967	down	2.869564	up
Os.8569.1.S1_at	LOC_Os04g48880	fatty acid hydroxylase, putative, expressed	7.0666585	down	3.1543837	up
**Secondary metabolism**						
Os.12161.1.S1_at	LOC_Os01g50760	polyprenyl synthetase, putative, expressed	2.1792645	down	1.5211341	up
Os.18305.1.S2_at	LOC_Os05g50550	polyprenyl synthetase, putative, expressed	4.09671	down	1.2781891	down
Os.22651.1.S1_at	LOC_Os04g56230	polyprenyl synthetase, putative, expressed	3.5511298	down	1.7434987	up
Os.18299.1.S1_at	LOC_Os07g07410	oxidoreductase, 2OG-Fe oxygenase family protein, putative, expressed	2.0075734	down	1.7679305	up
Os.28290.1.S1_at	LOC_Os02g47510	9-cis-epoxycarotenoid dioxygenase 1, chloroplast precursor, putative, expressed	3.8347073	down	5.148261	up
Os.57569.2.A1_s_at	LOC_Os08g04500	terpene synthase, putative, expressed	9.682299	down	1.2949013	up
OsAffx.13994.1.S1_at	LOC_Os04g27430	terpene synthase, putative	20.18755	down	1.5568593	up
OsAffx.28637.1.S1_x_at	LOC_Os07g27970	O-methyltransferase, putative, expressed	3.0486414	down	1.0420687	down
Os.11122.1.S1_at	LOC_Os08g37456	flavonol synthase/flavanone 3-hydroxylase, putative, expressed	3.2668943	up	1.0315864	up
Os.46813.1.S1_at	LOC_Os10g20610.1	laccase-15 precursor, putative, expressed	4.0983953	up	4.2176743	down
OsAffx.7606.1.S1_at	LOC_Os12g15680.1	laccase precursor protein, putative, expressed	6.6438375	up	12.205469	down
**Protein metabolism**						
Os.11946.1.S1_at	LOC_Os06g21380	OsCttP3 - Putative C-terminal processing peptidase homologue, expressed	14.64291	down	1.1892594	up
Os.50769.1.A1_at	LOC_Os06g03120	aspartic proteinase nepenthesin-2 precursor, putative	3.9677827	down	1.3502886	up
Os.52208.1.S1_at	LOC_Os11g45990	von Willebrand factor type A domain containing protein, putative, expressed	7.479702	down	1.1065638	down
Os.52261.1.S1_at	LOC_Os04g47360	OsPOP9 - Putative Prolyl Oligopeptidase homologue, expressed	6.0788016	down	1.0184413	down
Os.18819.1.S1_at	LOC_Os05g06660	OsSCP26 - Putative Serine Carboxypeptidase homologue, expressed	2.0857537	up	1.4075973	down
Os.4181.1.S1_at	LOC_Os01g67980	cysteine proteinase EP-B 1 precursor, putative, expressed	22.488976	up	8.29099	down
**Nucleic acid metabolism**						
Os.56275.1.S1_x_at	LOC_Os10g41100	CCT motif family protein, expressed	8.075855	down	2.332891	up
Os.57417.1.S1_s_at	LOC_Os05g50930	RNA polymerase sigma factor, putative, expressed	6.873295	down	1.0277363	up
Os.3407.1.S1_a_at	LOC_Os08g06630	RNA polymerase sigma factor, putative, expressed	3.5794642	down	2.7469041	up
Os.14820.1.S1_s_at	LOC_Os05g51150	RNA polymerase sigma factor, putative, expressed	2.5400774	up	1.2800328	up
**Photosynthesis**						
Os.12181.1.S1_s_at	LOC_Os11g13890	chlorophyll A–B binding protein, putative, expressed	4.180208	down	7.1355653	up
Os.12296.1.S1_at	LOC_Os03g39610	chlorophyll A–B binding protein, putative, expressed	6.138936	down	9.02072	up
Os.28216.2.S1_a_at	LOC_Os07g37550	chlorophyll A–B binding protein, putative, expressed	4.8379245	down	8.404701	up
Os.57519.1.S1_x_at	LOC_Os01g14410	early light-induced protein, chloroplast precursor, putative, expressed	4.234971	down	1.6210355	down
Os.7868.1.S1_at	LOC_Os01g17170	magnesium-protoporphyrin IX monomethyl ester cyclase,chloroplast precursor, putative, expressed	9.336843	down	6.4937716	up
**Defense**						
Os.2416.1.S1_a_at	LOC_Os01g71340	glycosyl hydrolases family 17, putative, expressed	4.742775	up	1.6272075	down
Os.418.1.S1_at	LOC_Os01g28450	SCP-like extracellular protein, expressed	12.021974	up	9.981392	down
Os.459.1.S1_at	LOC_Os03g46070	thaumatin, putative, expressed	3.5260322	up	5.110073	down
Os.49615.1.S1_at	LOC_Os03g45960	thaumatin, putative, expressed	3.8472998	up	8.540888	down
**Stress**						
**Drought stress**						
Os.12167.1.S1_at	LOC_Os02g44870	dehydrin, putative, expressed	2.9321172	down	1.1124701	up
Os.27642.1.S1_at	LOC_Os02g30320	drought-induced protein 1, putative, expressed	2.1810718	down	1.4512954	up
Os.54341.1.S1_at	LOC_Os12g43720	early-responsive to dehydration protein-related, putative, expressed	3.4949756	down	1.1637394	down
Os.6812.1.S1_at	LOC_Os10g21670	dehydration stress-induced protein, putative, expressed	20.037441	up	9.9064045	down
**GST**						
Os.22957.1.S1_at	LOC_Os01g72140	glutathione S-transferase, putative, expressed	7.938599	up	12.89018	down
Os.40000.1.S1_at	LOC_Os10g38600	glutathione S-transferase GSTU6, putative, expressed	3.4470932	up	7.7719364	down
Os.46634.1.S1_at	LOC_Os10g38489	glutathione S-transferase GSTU6, putative, expressed	2.544139	up	5.0398207	down
Os.46635.1.S1_x_at	LOC_Os10g38350.1	glutathione S-transferase, putative, expressed	2.723984	up	7.052062	down
**Heat stress**						
Os.26059.1.S1_at	LOC_Os03g20730	chaperone protein dnaJ, putative, expressed	2.3602993	down	1.4174172	up
Os.49277.1.S1_at	LOC_Os01g53020	heat shock protein DnaJ, putative, expressed	2.5835004	down	5.9116116	up
Os.5648.1.S1_at	LOC_Os01g01160	heat shock protein DnaJ, putative, expressed	4.7269297	down	1.9027811	up
Os.57477.1.S1_x_at	LOC_Os11g47760.1	DnaK family protein, putative, expressed	2.0001137	down	1.4022813	up
OsAffx.11879.1.S1_at	LOC_Os02g03570	hsp20/alpha crystallin family protein, putative, expressed	2.594732	up	3.2607677	down
Os.11941.2.S1_at	LOC_Os09g35790	HSF-type DNA-binding domain containing protein, expressed	2.0028756	down	1.1283132	up
OsAffx.26649.1.S1_x_at	LOC_Os04g57880	heat shock protein DnaJ, putative, expressed	7.3370676	up	7.996968	down
**Oxidation-reduction**						
Os.23290.1.S1_at	LOC_Os10g40960	oxidoreductase, 2OG-Fe oxygenase family protein, putative, expressed	2.322114	up	1.8621931	down
Os.25496.1.S1_at	LOC_Os11g07930	oxidoreductase, short chain dehydrogenase/reductase family domain containing family, expressed	3.465435	up	17.474422	down
Os.7392.2.S1_x_at	LOC_Os01g62880	oxidoreductase, aldo/keto reductase family protein, putative, expressed	2.1068213	up	2.3765166	down
Os.9230.1.S1_x_at	LOC_Os10g35370	oxidoreductase, short chain dehydrogenase/reductase family domain containing family, expressed	2.0683372	down	4.2801576	up
Os.11266.1.S1_at	LOC_Os01g09830	OsGrx_A2 - glutaredoxin subgroup III, expressed	4.5280557	up	16.795298	down
Os.12100.1.S1_at	LOC_Os07g29410	thioredoxin, putative, expressed	2.4418237	down	2.615967	up
Os.36648.1.S1_x_at	LOC_Os01g13480	glutaredoxin, putative, expressed	3.831749	down	1.0782503	down
OsAffx.15537.1.S1_at	LOC_Os06g21550	thioredoxin domain-containing protein 17, putative, expressed	4.0799003	up	2.8130107	down
**Plant hormone**						
**Auxin**						
OsAffx.14380.1.S1_s_at	LOC_Os04g52670.1	OsSAUR21 - Auxin-responsive SAUR gene family member, expressed	2.4365497	up	1.091372	up
Os.12501.1.S1_at	LOC_Os01g55940	OsGH3.2 - Probable indole-3-acetic acid-amido synthetase, expressed	2.102113	up	2.3879192	down
Os.18493.1.S1_at	LOC_Os09g37480	OsSAUR53 - Auxin-responsive SAUR gene family member, expressed	2.7041986	down	2.0083132	up
Os.19952.1.S1_at	LOC_Os01g09450	OsIAA2 - Auxin-responsive Aux/IAA gene family member, expressed	2.3135202	up	1.126054	down
Os.56924.1.S1_at	LOC_Os05g41420	auxin-induced protein 5NG4, putative, expressed	2.6951323	down	2.5001767	up
**Jasmonic Acid**						
Os.15829.1.S1_at	LOC_Os02g10120	lipoxygenase, putative, expressed	16.1165	down	4.611044	up
Os.6863.1.S1_at	LOC_Os12g14440	Jacalin-like lectin domain containing protein, putative, expressed	65.412315	down	1.8992921	up
**Gibberellin**						
Os.27673.1.S1_at	LOC_Os03g14730	gibberellin receptor GID1L2, putative, expressed	2.4927118	down	1.7543458	up
Os.30608.1.S1_at	LOC_Os02g41954	gibberellin 2-beta-dioxygenase 7, putative, expressed	2.1261473	down	2.3699095	up
Os.33885.1.S1_at	LOC_Os01g06220	gibberellin receptor GID1L2, putative, expressed	3.5340044	up	9.710723	down
**Ethylene-responsive**						
OsAffx.12799.1.S1_s_at	LOC_Os03g09170	ethylene-responsive transcription factor, putative, expressed	4.337239	down	1.7307606	up
OsAffx.12775.1.S1_at	LOC_Os03g07940	AP2 domain containing protein, expressed	2.0415235	up	1.8989204	down
**Cell wall**						
Os.158.1.S1_at	LOC_Os10g02070	peroxidase precursor, putative, expressed	2.1756594	down	4.579428	up
Os.2373.1.S1_at	LOC_Os10g40710	expansin precursor, putative, expressed	3.3498163	down	3.2064276	up
Os.5034.1.S1_at	LOC_Os07g47990	peroxidase precursor, putative, expressed	3.2906973	down	3.9200315	up
Os.57547.1.S1_at	LOC_Os07g48050	peroxidase precursor, putative, expressed	16.27228	up	13.841287	down
OsAffx.17389.1.S1_s_at	LOC_Os08g37930	beta-expansin precursor, putative, expressed	4.7917747	up	1.8499234	down
Os.5087.1.S1_at	LOC_Os11g03780.1	alpha-N-arabinofuranosidase, putative, expressed	2.0912318	up	1.0629448	down
OsAffx.26676.1.S1_at	LOC_Os05g01370	polygalacturonase inhibitor precursor, putative, expressed	2.1363134	up	1.4509802	down
**Signal ransduction**						
**CIPK**						
Os.41019.1.S1_at	LOC_Os01g35184.1	CIPK8	2.0357978	down	1.1911937	up
Os.17787.1.S1_at	LOC_Os07g48090	CIPK29	3.334685	down	3.260447	up
Os.19815.1.S1_at	LOC_Os03g43440	CIPK7	2.9495628	down	3.3653092	up
**MAPK**						
Os.11617.1.S1_at	LOC_Os05g49140	CGMC_MAPKCMGC_2.8 - CGMC includes CDA, MAPK, GSK3, and CLKC kinases, expressed	2.0979733	down	2.1305962	up
Os.27170.1.S1_at	LOC_Os01g50400	STE_MEKK_ste11_MAP3K.5 - STE kinases include homologs to sterile 7, sterile 11 and sterile 20 from yeast, expressed	3.7302904	down	2.896405	up
Os.5940.1.S1_at	LOC_Os01g50370	STE_MEKK_ste11_MAP3K.4 - STE kinases include homologs to sterile 7, sterile 11 and sterile 20 from yeast, expressed	6.0885477	down	2.018108	up
**Calcium sensor**						
Os.2216.1.S1_at	LOC_Os01g59530	OsCML1 - Calmodulin-related calcium sensor protein, expressed	2.9643536	up	2.7207763	down
Os.49658.1.S1_at	LOC_Os05g45810	calcineurin B, putative, expressed	3.1708822	down	1.2174544	up
Os.8862.1.S1_at	LOC_Os09g28510	EF hand family protein, putative, expressed	2.0125606	up	1.5394444	down
Os.8889.1.S1_at	LOC_Os05g50180	OsCML14 - Calmodulin-related calcium sensor protein, expressed	3.8419423	down	2.0545144	up
**Phosphatase**						
Os.12150.1.S1_at	LOC_Os04g33080	protein phosphatase 2C, putative, expressed	2.380953	down	1.4617312	up
Os.12535.1.S1_at	LOC_Os01g52230	phosphoethanolamine/phosphocholine phosphatase, putative, expressed	5.0726867	down	1.2910246	down
Os.49226.1.S1_at	LOC_Os12g38750	nucleotide pyrophosphatase/phosphodiesterase, putative, expressed	3.5158448	up	1.9599011	down
Os.49352.1.S1_at	LOC_Os11g15570	Ser/Thr protein phosphatase family protein, putative, expressed	2.0299246	up	1.8765	down
Os.5690.1.S1_at	LOC_Os01g46760	protein phosphatase 2C, putative, expressed	2.3383155	down	1.2241448	up
**Transporters**						
**Potassium transporter**						
Os.11262.2.S1_x_at	LOC_Os04g32920	HAK1	1.6623486	up	1.3617935	down
OsAffx.3292.1.S1_s_at	LOC_Os03g21890	potassium transporter, putative, expressed	1.89058	down	1.1096667	up
Os.38354.1.S1_x_at	LOC_Os01g11250	potassium channel KAT1, putative, expressed	1.7454123	down	1.2604069	up
**ABC transporter**						
Os.10975.1.S1_at	LOC_Os11g39020	ABC transporter, ATP-binding protein, putative, expressed	5.1243515	down	1.092812	up
Os.11616.1.S1_at	LOC_Os02g36570	ABC1 family domain containing protein, putative, expressed	3.1116002	down	1.6521326	up
**Sodium transporter**						
Os.19896.2.S1_x_at	LOC_Os06g48800	OsHKT2;4 - Na+ transporter, expressed	6.008856	down	1.069684	down
Os.57530.1.S1_x_at	LOC_Os01g34850	OsHKT2;3 - Na+ transporter, expressed	3.2722178	down	1.0480005	down
**Sulfate transporter**						
Os.18597.1.S1_at	LOC_Os03g09930	sulfate transporter, putative, expressed	10.878617	down	1.1813235	down
Os.10754.1.S1_at	LOC_Os03g06520	sulfate transporter, putative, expressed	2.6930013	down	2.0457704	up
**Lipid transporter**						
Os.20387.1.S1_at	LOC_Os03g14642	LTPL107 - Protease inhibitor/seed storage/LTP family protein precursor, expressed	3.3959548	up	1.7493165	down
Os.24103.1.A1_at	LOC_Os07g18990	LTPL40 - Protease inhibitor/seed storage/LTP family protein precursor, expressed	2.3825653	up	1.1182239	down
Os.36913.1.S1_at	LOC_Os10g40530	LTPL146 - Protease inhibitor/seed storage/LTP family protein precursor, expressed	3.1217232	down	5.417366	up
Os.38235.2.S1_at	LOC_Os04g52260	LTPL124 - Protease inhibitor/seed storage/LTP family protein precursor, expressed	5.2148013	down	6.255761	up
Os.6838.1.S1_at	LOC_Os10g40510	LTPL144 - Protease inhibitor/seed storage/LTP family protein precursor, expressed	3.2739449	down	7.4096494	up
**Peptide transporter**						
Os.32686.1.S1_at	LOC_Os01g65110	POT family protein, expressed	2.889076	up	3.8825707	down
Os.34161.1.S1_at	LOC_Os06g03700	oligopeptide transporter, putative, expressed	2.7034025	up	3.4326303	down
Os.45923.1.S1_at	LOC_Os01g37590	peptide transporter PTR2, putative, expressed	2.8099432	down	3.7542121	down
Os.52974.1.S1_at	LOC_Os04g50940	peptide transporter PTR2, putative, expressed	2.4533195	up	3.851256	down
Os.9303.1.S1_at	LOC_Os02g46460	peptide transporter PTR2, putative, expressed	5.1835947	down	19.712648	up
**Metal transporter**						
Os.1193.1.S1_at	LOC_Os04g43070	ammonium transporter protein, putative, expressed	3.5124	down	1.6415328	up
Os.26807.1.S1_at	LOC_Os01g12210	aluminum-activated malate transporter, putative, expressed	4.311443	up	1.5286913	down
Os.27805.1.S1_at	LOC_Os03g48000	CorA-like magnesium transporter protein, putative, expressed	7.6011996	down	2.93601	up
Os.54471.1.S1_at	LOC_Os08g10630	metal cation transporter, putative, expressed	2.1970346	down	1.2260257	up
Os.6864.1.S1_at	LOC_Os01g61070	heavy metal-associated domain containing protein, expressed	3.188327	up	2.0711086	down
**Transcription factor**						
**Myb TF**						
Os.49829.1.S1_at	LOC_Os02g40530.1	MYB family transcription factor, putative, expressed	4.539408	down	2.2878835	up
Os.10901.1.S1_a_at	LOC_Os08g06110	MYB family transcription factor, putative, expressed	26.226946	down	1.342114	up
Os.18955.1.S1_at	LOC_Os06g51260	MYB family transcription factor, putative, expressed	16.628183	down	1.4534045	up
Os.3340.1.S1_a_at	LOC_Os06g24070	myb-like DNA-binding domain containing protein, expressed	8.957898	down	13.670861	up
Os.8149.1.S1_at	LOC_Os02g46030	MYB family transcription factor, putative, expressed	42.721607	down	1.0932941	up
**Zinc finger TF**						
Os.12805.1.S1_at	LOC_Os04g41560	B-box zinc finger family protein, putative, expressed	12.830358	down	10.982284	up
Os.23145.1.S1_at	LOC_Os08g03310	zinc finger family protein, putative, expressed	5.089536	down	3.4042013	up
Os.3399.1.S1_at	LOC_Os09g06464	CCT/B-box zinc finger protein, putative, expressed	9.954306	down	2.2954476	up
Os.4184.1.S1_at	LOC_Os02g15350	dof zinc finger domain containing protein, putative, expressed	6.1414027	up	5.342717	down
Os.10583.1.S1_at	LOC_Os06g04920	zinc finger family protein, putative, expressed	14.976689	up	6.472622	down
**bZIP TF**						
Os.16025.1.S1_s_at	LOC_Os06g39960	bZIP transcription factor domain containing protein, expressed	3.1624238	down	1.4623934	down
Os.12134.1.S1_at	LOC_Os04g45810	homeobox associated leucine zipper, putative, expressed	16.334263	down	1.1461058	up
Os.27388.1.S1_s_at	LOC_Os10g01470	homeobox associated leucine zipper, putative, expressed	2.4151278	up	3.0738394	down
**HLH TF**						
Os.19229.1.S1_a_at	LOC_Os03g43810	helix-loop-helix DNA-binding domain containing protein, expressed	2.5253093	down	1.4796277	up
Os.27587.1.S1_at	LOC_Os10g40740	helix-loop-helix DNA-binding domain containing protein, expressed	2.0795941	down	1.2293708	down
Os.4548.1.S1_at	LOC_Os04g52770	helix-loop-helix DNA-binding domain containing protein, expressed	3.0124726	up	3.9773142	down
Os.46956.1.S1_at	LOC_Os01g50940	helix-loop-helix DNA-binding domain containing protein, expressed	9.380022	down	3.0063295	up

### K-means Cluster Analysis

**Figure 6:**
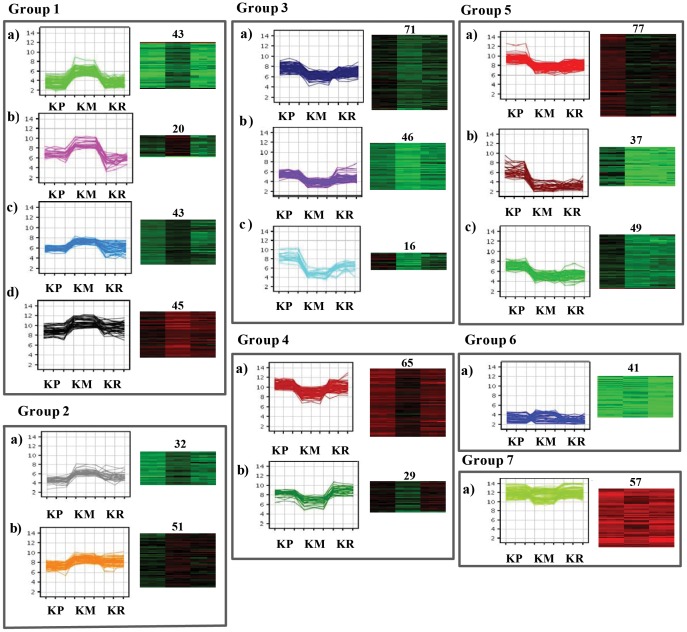
K-means cluster analysis of differentially expressed genes. All the genes categorized into 20 clusters and again grouped together to make 7 groups, based on their similar expression patterns but different expression amplitudes. The normalized log transformed signal values were plotted for all the conditions. The number of genes in the clusters is indicated upper side of the heatmap.

To elucidate potassium-specific gene expression profiles, co-regulated genes were further analysed by K-Means Cluster analysis. Generally, K-means cluster analysis is based on the assumption that genes involved in either a similar function or a common pathway will have similar expression profiles and can likely to be grouped together. The analysis was performed for genes that were significantly expressed during potassium starvation. A total of 722 DEG under potassium-deficient conditions were clustered into 16 primary clusters and were then grouped into 7 different groups based on significant changes in expression pattern (Figure 6). Group 1, the largest group, consisted of 151 genes that were further divided into four sub-groups (i.e. 1A, 1B, 1C and 1D). All the genes in this group exhibited high expression levels under potassium-deficient conditions (KM), while their expression was low in both potassium-sufficient (KP) and potassium resupply (KR) conditions. Stress-related genes such as oxidoreductase, GST, heat shock protein and a few genes related to metabolism such as oligosaccharyl transferase, terpene synthase, ribulose 1,5 biphosphate were included in this group. 32 and 51 genes from group 2 a and 2 b, respectively, showed similar expression pattern as group 1 under KM condition, but they exhibited slightly elevated expression during potassium resupply (KR) compared to K^+^ plus (KP) conditions. This group included genes related to plant growth, plant development, and transcription factors. In group 3 (133 genes) and group 4 (94 genes), the overall expression pattern of genes was similar, but the difference in expression levels of genes under KM and KR was greater in group 4 as compared to group 3. Group 3 included 133 genes; all of them showed elevated expression in KP and low expression in KM. After resupply of potassium, they regained expression levels comparable to KP. Most of the photosynthesis, signal transduction, and transporter-related genes along with flavonol synthase, expansin precursor, and RNA polymerase sigma factor, comprised group 3 and 4. 163 genes of group 5 had elevated expression in K^+^-sufficient (KP) conditions and constant expression in K^+^-deficient (KM) and K^+^-resupply condition (KR). CHIT10, WRKY 50, WRKY 65, a MYB family transcription factor were also contained in this group. Group 6 genes were not differentially expressed in any of the three conditions tested and maintained a constant low level of expression. On the other hand, genes placed in group 7 showed high expression in all three conditions.

### Genes Involved in Metabolism

A large number of DEG was found to be involved in metabolic processes, particularly primary metabolism (carbohydrate and lipid), secondary metabolism, protein and nucleic acid metabolism. Potassium is a crucial ion for plants as it acts as a cofactor for various enzymes in metabolic pathways [10]. From this gene expression analysis, many genes related to metabolism showed altered expression in potassium-deficient conditions (KM). Upon resupply of potassium, the expression was restored to similar levels as seen in K^+^-sufficient (KP) conditions. As mentioned earlier, 18% of differentially regulated genes were involved in various metabolic and biosynthetic processes. Among these 18% of genes, 30 genes are related to carbohydrate metabolism, of which 10 genes were downregulated in KM condition and upregulated upon external resupply of potassium (KR). Another 14 genes showed the reverse trend of upregulation in KM condition and downregulation in KR condition (Table S5). Carboxyvinyl-carboxyphosphonate phosphorylmutase (LOC_Os12g08760) showed downregulation in potassium-deficient conditions, as well as glyoxalase (LOC_Os07g06660), pyruvate kinase (LOC_Os11g10980), and lactate/malate dehydrogenase (LOC_Os07g43700). UDP-glucoronosyl and UDP-glucosyl transferases (LOC_Os01g08440, LOC_Os02g11640), and a glycosyltransferase (LOC_Os01g07530) showed downregulation in both deficient and resupply condition. Phosphoglycerate kinase (LOC_Os06g45710) and indole-3-glycerol phosphate synthase (LOC_Os09g08130) were up- and downregulated in KM and KR condition, respectively. Alpha-amylase (LOC_Os09g28430, LOC_Os02g52700) showed the highest expression in deficient condition (Table 2).

Eleven of the lipid metabolism-associated genes were downregulated in potassium-deficient conditions and showed elevated expression in resupply conditions (Table S5). Beta-amylase (LOC_Os03g04770), omega-3 fatty acid desaturase (LOC_Os03g18070), and lipase (LOC_Os04g56240) exhibited low expression in deficient condition and high expression upon resupply. Three genes related to lipid metabolism showed the reverse trend and were upregulated in KM and downregulated in KR condition. A number of other genes related to metabolism such as transferases (10 genes), dehydrogenases (5 genes) other kinases/phosphatases (4 genes) were also differentially regulated (Table S5).

### Genes Involved in Secondary Metabolism

Eleven K^+^-responsive genes related to secondary metabolism were also downregulated upon KM treatment and upregulated in KR conditions. This category included many enzymes such as two polyprenyl synthetases (LOC_Os05g50550, LOC_Os04g56230) and a protein containing a FAD-binding domain (LOC_Os02g51080). Seven genes encoding flavonol synthase (LOC_Os10g40934), dihydroflavonol-4-reductase (LOC_Os07g41060), and laccase precursor protein (LOC_Os12g15680, LOC_Os10g20610) showed upregulation in KM conditions and downregulation in KR conditions (Table 2).

### Nucleic Acid and Protein Metabolism

In potassium-deficient conditions, the majority of DEG not only belonged to carbohydrate and lipid metabolism, but several genes were identified that are annotated as being involved with nucleic acid and protein metabolism. Among 22 genes associated with nucleic acid metabolism, only 8 showed downregulation in KM condition and elevated expression in KR condition, whereas the remaining exhibited reversed expression pattern (Table S5). Transcripts presenting this profile encoded, for example, two AMP deaminases (LOC_Os05g28180, LOC_Os07g46630); endonuclease/exonuclease/phosphatase domain containing (LOC_Os01g58690); RNA polymerase sigma factor (LOC_Os08g06630, LOC_Os05g51150); and 3′–5′ exonuclease (LOC_Os04g14810). Most of the protein metabolism-related transcripts shown in Table 2 were downregulated upon potassium treatment, and their expression increased upon KR treatment. The putative C-terminal processing peptidase OsCttP3 (LOC_Os06g21380), the putative prolyl oligopeptidase OsPOP9 (LOC_Os04g47360), aspartic proteinase nepenthesin-2 (LOC_Os06g03120), and 8 other genes were included in this category.

### Genes Related to Photosynthesis and Plant growth

Photosynthesis is a process by which green plants capture light energy to synthesize organic compounds from carbon dioxide and water. Seventeen photosynthesis-related genes were expressed differentially under KM and KR conditions. Surprisingly, all 17 genes were downregulated in KM condition and upregulated in KR condition. Some of these genes (9) encoded chlorophyll a/b binding protein, photosystem I reaction centre subunit (LOC_Os09g30340), and magnesium-protoporphyrin IX monomethyl ester cyclase (LOC_Os01g17170) (Table 2). Sixteen transcripts (9 upregulated and 7 downregulated) that showed altered expression in potassium-deficient conditions relative to their expression under normal growth conditions were related to plant growth and development. The expression of these genes was reversed upon resupply of potassium (Table S5).

### Transcription Factors and Related Genes

In higher plants, gene expression in response to various stresses is regulated by transcription factors [26]–[28]. In many biological pathways, transcription factors are considered to be master regulators, since they can control the expression of suites of multiple genes at a given time in response to a particular stress condition. Our microarray results showed that 55 genes encoding transcription factors were induced under potassium deficiency (Table S5). Among the 55 induced genes, 21 genes belong to the zinc finger family and 13 to the MYB gene family. Another 4 genes are bZIP (basic region/leucine zipper motif) transcription factors, and 4 other genes are HLH (Helix-loop-Helix) transcription factors, 2 are annotated as ethylene-responsive (ERF) transcription factors. Of the 19 zinc finger transcription factors, only 5 were upregulated in KM condition and downregulated upon KR condition, whereas the remaining 14 showed the reverse expression pattern. Similar expression patterns were observed for all other categories of transcription factors. Some of the MYB family transcription factors (LOC_Os02g46030, LOC_Os06g51260, LOC_Os08g06110) were strongly downregulated under deficient conditions and upregulated upon resupply of potassium (Table 2).

### Transporters

Potassium deficiency affected the expression of a large number of transcripts encoding transporters representative of several distinct protein families. Similar to transcription factors, some transporters also showed downregulation under potassium deficiency and upregulation upon resupply. Of the 40 differentially expressed transporters, 9 were metal transporters; 7 were peptide transporters; and 5 were lipid transporters. In this study, sodium transporters (OsHKT2;3 and OsHKT2;4 2 ), sulphate transporters (2 genes), and ABC transporters (2 genes) were highly downregulated in KM conditions and upregulated under KR conditions (Table 2). Thirteen other transporters (annotated as aquaporins, MATE efflux transporters, and AAA-type ATPases) also displayed similar expression pattern as described above. Surprisingly, we did not find any potassium transporters that showed differential expression in our microarray data with the criteria of more than 2-fold change. But when the fold change criterion was reduced to 1.5, two potassium transporters (OsHAK1), one potassium channel (OsKAT1) and one putative potassium transporter were found to be differentially expressed (Table S6).

### Signal Transduction Related Genes

Based on the available literature for analyses of gene expression changes in response to various nutrient deficiencies, several proteins involved in signal transduction networks such as calcium sensors, kinases, and phosphatases were found to be differentially regulated under those conditions [24], [25], [29], [30]. Kinases and phosphatases are involved in a large number of distinct signaling pathways, such as the signal transduction pathways controlling cell growth, differentiation, and death. In our analysis, 31 differentially expressed genes were classified as signal transduction-related genes. Three CIPKs (CIPK7, CIPK8 and CIPK29) and three MAPKs display distinct patterns of downregulation and upregulation in potassium-deficient and resupply conditions, respectively (Table S5). Among the other signaling related genes, 6 phosphatases, 2 PP2Cs (LOC_Os01g46760, LOC_Os04g33080), and a phosphocholine phosphatase exhibited low expression under KM condition and elevated expression under KR condition, whereas one of the Ser/Thr protein phosphatases and two of the pyrophosphatases exhibited the reverse expression pattern. Some of the kinases such as a CDPK (LOC_Os03g48270) and the serine/threonine protein kinase SNT7 (LOC_Os05g47560) also showed downregulation in KM condition, but BRASSINOSTEROID INSENSITIVE 1 (LOC_Os11g31530) was upregulated 12-fold under KM conditions and down regulated 10-fold under KR conditions (Table 2).

### Stress Related Genes

Reactive oxygen species (ROS) are known to be involved in signaling pathways specific to low potassium stress conditions [31], [32]. Oxidoreductase and glutathione S-transferase (GST) play important roles during oxidative stress in plants [33]. In our study, a total of 50 genes grouped as stress-related were differentially expressed under low potassium conditions. These included 28 upregulated and 22 downregulated genes. Among these, 5 oxidoreductase genes and 4 GST were identified that showed upregulation in KM conditions and downregulation in KR conditions (Table S5), which we view as unsurprising given the well established role of potassium in stress responses [34]–[36]. A total of 11 heat stress-related genes (HSPs) and 5 dehydration-related genes were identified. Most of the HSPs (9 genes) were downregulated in potassium-deficient conditions, and the expression pattern was reversed upon resupply of potassium. Other drought-responsive genes showed a qualitatively similar expression pattern (Table S2).

### Cell Wall and Cell Death Related

Among 12 cell wall-related genes identified in this study, 9 genes encoding peroxidases (LOC_Os07g48050, LOC_Os01g73170, LOC_Os03g13210), expansin (LOC_Os01g60770), and alpha-N-arabinofuranosidase (LOC_Os11g03780) were upregulated, and another 2 peroxidases (LOC_Os10g02070, LOC_Os07g47990) and an expansin (LOC_Os10g40710) were down regulated during potassium deficiency and showed reversal of expression upon resupply (Table 2).

Cell death-related transcripts constituted only a small category in the microarray results. This category contained 7 genes, including 5 genes encoding ubiquitin, which mostly showed upregulation under low potassium conditions (KM) (Table S5).

### Phytohormones and Defense Related Genes

Changes in potassium supply affect the transcription of genes related to phytohormones like jasmonic acid (JA), auxin, gibberellin and others [5], [37]. Seven genes were found to be related to auxin, such as *OsSAUR21*, *OsSAUR53*, *OsIAA2* (Table 2, S5). Interestingly, two jasmonic acid-related genes, lipoxygenase (LOC_Os02g10120) and jacalin-like lectin (LOC_Os12g14440) were upregulated 16- and 65-fold in potassium-deficient conditions, respectively. Their expression levels declined rapidly after resupply. Two GA-related genes also showed similar expression patterns as the jasmonic acid related genes, however GID1, exhibited the opposite expression pattern.

Thirteen genes related to plant defense were also differentially expressed, 7 genes were downregulated in KM condition and upregulated upon resupply (KR). The remaining 6 genes had a reverse expression pattern (i.e. upregulated in KM and downregulated in KR conditions). This category include genes encoding glycosyl hydrolase (LOC_Os05g31140, LOC_Os01g71340), cysteine protease 1 (LOC_Os03g54130), thaumatin (LOC_Os03g45960, LOC_Os03g46070), and WIP3 (LOC_Os11g37950).

### cis-Regulatory Element Analysis of Potassium Deficiency Responsive Genes

When analysing the expression of a group of genes that respond similarly to a particular condition, it is anticipated that these genes might have some common features or elements driving their expression. The promoters of co-expressed genes might share some common regulatory elements or be regulated by a common set of transcription factors. Thus, the identification of shared *cis*-regulatory elements in the promoter regions of genes co-expressed in response to altered levels of potassium availability could provide new insights into the transcriptional regulatory networks that are triggered by potassium deficiency in plants. To identify common *cis*-regulatory elements, 1 kb regions directly upstream of potassium-responsive genes in *Arabidopsis* and rice were analysed in PLACE database [38], [39]. Genes that exhibited high levels of expression under potassium deficiency in this study and earlier studies on *Arabidopsis* including CIPK9 [40], HAK5 [41], peroxidase, glycosyl hydrolase, AP2 domain-containing transcription factor, rice alpha-amylase, glycosyl hydrolase, peroxidase, potassium transporter HAK1, GST, dehydration stress-induced protein, and ethylene-responsive transcription factor were compared. A total of 31 common *cis*-regulatory elements were found, including ABRELATERD1 (ACGTG), ARR1AT (NGATT), CAATBOX1 (CAAT), DOFCOREZM (AAG), GATABOX (GATA), GT1CONSENSUS (GRWAAW), POLLEN1LELAT52 (AGAAA), TAAAGSTKST1 (TAAAG), WRKY71OS (TGAC) and many others that were prominently present in the 1 kb putative regulatory regions of the aforementioned genes. A list of all of the 31 *cis*-regulatory elements with their sequences is provided in Table S7.

### Comparative Analysis of Rice and Arabidopsis in Potassium and Other Nutrient Deprivation

Transcriptome data from this study were compared to earlier published results on the transcriptome response to potassium deficiency in *Arabidopsis* and rice, particularly an earlier transcriptomic profile of 2 week old rice roots grown under potassium-deficient conditions [37]. However, the transcriptome profile of potassium-deficient plants is comparatively far better characterized in *Arabidopsis*, where both two week old shoot and root tissues were analyzed under potassium deprivation [5], [42]. We compared the dataset; we obtained from seedlings grown under potassium-deficient conditions under these two published transcriptome datasets. In earlier reports, far fewer genes were reported to be differentially regulated with a 2-fold or greater expression change in response to potassium deficiency in both rice (722 in rice whole seedling [this study] versus 356 in rice root [37]) and *Arabidopsis* (119 in shoot, 299 in root [5]) (Table 3). In an attempt to identify the similarities and differences among potassium-responsive genes in rice and *Arabidopsis*, we categorised and compared 356 DEG from rice root [37] with 722 DEG from whole rice seedling (this study) and 418 genes from *Arabidopsis* shoot and root transcriptome [5] (Figure S1). This comparative analysis revealed that the majority of DEG was related to metabolism and signal transduction in response to potassium deficiency in both rice and *Arabidopsis* (Figure S1). The percentage of genes related to defense and cell wall were significantly greater in number in *Arabidopsis* than rice. In whole rice seedlings, the number of differentially expressed transcription factor genes were greater (this study, Figure S1) compared to the roots of potassium-deficient rice seedlings [37]. The present transcriptome profile of potassium-deficient rice seedlings was further compared to transcriptome profiles of rice under other nutrient deprivation (i.e. nitrogen, phosphorus, and iron) (Figure S2). From the above studies, it was inferred that genes differentially expressed in response to deprivation of distinct nutrients were associated with various metabolic processes and stress adapatations and were classified as transporters, transcription factors, and signal transduction components. This suggest that there might be a functional overalap of these genes in responses to deprivation of nutrients other than potassium in both rice and *Arabidopsis*.

**Table 3:** Differentially expressed genes from present microarray data and data obtained from 2 week K^+^-starved *Arabidopsis* shoot-root (Armengaud et al. 2004), roots of rice plants after 6 h, 3 days and 5 days (Ma et al. 2012).

Study	(Armengaud et al. 2004)	(Ma et al. 2012)	Our data
Organ	*Arabidopsis* shoot and root	Rice root	Rice seedling
Cutoff value	FDR%<1	Fold Change ≥2 and P-value ≤0.05	Fold Change ≥2 and P-value ≤0.05
Regulated Gene	116 and 299	356	722

### Relationship of Potassium Ion Response Related Genes with Abiotic Stresses

We analysed potassium responsive DEGs (mainly related to metabolism, signal transduction and stress) under abiotic stresses like cold, drought, heat and salt stress, using Genevestigator database for their transcript status. Most of the genes showed differential expression pattern under these stresses. Metabolism related carboxyvinyl-carboxyphosphonate phosphorylmutase (LOC_Os12g08760), heparanase (LOC_Os06g08090), UDP glucoronosyl (LOC_Os01g08440) showed downregulation in all stresses except heat stress. But phosphoglycerate kinase (LOC_Os06g45710) and beta-amylase (LOC_Os03g04770) were upregulated in all stress conditions (Figure S3A). Genes related to signal transduction, such as OsCML14 (LOC_Os05g50180) and BRI I, (LOC_Os11g31530) showed an upregulation in both cold and drought stresses whereas no differential expression was found in heat and salt stresses. MAP3K4 (LOC_Os01g50370) expression was elevated in response to above mentioned four stresses. In contrast, EF-hand containing protein (LOC_Os09g28510) was showing downregulation in stressed state (Figure S3B). Early responsive to dehydration protein (LOC_Os12g43720), oxidoreductase (LOC_Os11g07930), HSP (LOC_Os2g54140), HSF (LOC_Os02g35960) genes were downregulated in cold and heat stresses while upregulated under drought stress. Dehydrin (LOC_Os02g44870) showed elevated expression under cold, drought, heat and salt stresses whereas 2-oxoglutarate (LOC_Os10g40960) and oxidoreductase (LOC_Os02g53510) were downregulated under these conditions (Figure S3C).

## Discussion

Nutritional deficiencies alter the physiology and metabolism of plants during both short-term and long-term periods of growth. In response to nutrient deficiency, plants activate a plethora of genes and gene networks encoding a large number of proteins involved in acclimation and adaptation. Significant research has contributed to our understanding of the uptake, distribution and homeostasis of potassium ions in *Arabidopsis* by employing advanced tools for genetics, biochemistry, cell biology, and physiology. Our knowledge of adjustment and adaptation to short-term and long-term nutrient deficiency is still insufficient to establish a basic model of how plants manage to thrive physiologically despite nutrient deficiencies in soil. As mentioned in the introduction, research related to potassium nutrition in plants has drawn the attention of several leading research groups who seek to enhance our understanding of the mechanisms of plant tolerance to potassium deficiency. In this study, we have contributed to this effort by performing detailed transcriptomic profiling of whole rice seedlings exposed to short-term (5 days) potassium deprivation.

It is quite possible that different plant species might have different mechanism to absorb, transport, distribute, and utilize potassium [4], [6], [7], [43]–[45]. Biomass is one important parameter to measure plant growth under low nutrient conditions, and it is a significant criterion to judge the crucial role played by particular nutrient in plant growth and development.

In order to complement our transcriptomic analysis of rice seedlings, we also performed a physiological experiment to determine the growth symptoms of potassium deficiency. The experimental results indicate that rice grew significantly more poorly under low potassium conditions than it did under higher concentrations of potassium that we believe are more reflective of optimal growth conditions for rice. This effect is clearly seen in the qualitative and quantitative assessment of seedling biomass under both conditions (Figure 1A, B, C). At the molecular level, the content of potassium ions in plants grown in low potassium media was also appreciably lower than the potassium content of plants grown in media with sufficient levels of potassium, which demonstrates that rice seedlings are indeed able to take up higher levels of potassium when external levels are sufficiently higher and further establishes a direct link between potassium supply and plant growth (Figure 1D). Similar results were previosuly described in *Arabidopsis* plants starved of potassium [5]. Analysis of the growth of *Arabidopsis* under long-term potassium starvation (14 days) by Armengaud et al. (2004) [5] revealed chlorosis (bleaching) of older leaves, as well as a reduction in lateral root growth of seedlings. In contrast to these reported results in *Arabidopsis*, we observed an obvious reduction in the growth of rice seedlings by the fifth day of growth on low potassium containing media without any significant signs of chlorosis or bleaching of seedling leaves. We also observed a very similar, severe impairment of growth in rice seedlings maintained on potassium-deficient media for longer period of time (i.e. 14 days; data not shown). After observing growth differences of rice seedlings exposed to short-term or long-term potassium deprivation, we decided to investigate gene expression profiles under short-term (5 days) potassium starvation.

During the preparation of this manuscript, we discovered a report on the transcriptome profile of rice roots deprived of potassium [37]. The major difference between this study and our study is that we chose to use whole seedlings rather than just roots. Moreover, this study is more comprehensive due to our transcriptional profiling of seedlings resupplied with potassium after short-term potassium deprivation. These growth conditions closely mimicked a previous study by Armengaud et al. 2004 [5]. The usage of higher level of stringency (i.e. 2-fold or greater change) and false discovery rate (FDR) in the present study also strengthens the reliability of our data on differential gene expression. Despite several differences, some of our findings are consistent with differentially expressing genes previously reported by Ma et al. 2012 [37].

The most significant contribution of the present work is the identification of genes implicated in the growth and development of rice seedling exposed to the short-term stress of potassium deficiency and the resupply of potassium to deprived seedlings. This study provides a platform for the investigation of the mechanisms of regulatory responses to low potassium conditions. From our microarray analysis, a large number of genes (722) were found to be differentially expressed during potassium deprivation and resupply (1867 DEG). Only 307 of the affected genes, however, exhibited reversal of gene expression (i.e. restoration of comparable expression levels as seen during optimal growth conditions) upon resupply of potassium to the deficient media (Figure 2B), therefore this study clearly identifies a subset of the differentially expressed genes that are specifically expressed only during potassium deprivation. This difference in expression is also seen in the results of our PCA analysis, where the three biological replicates of the potassium resupply treatment were more similar to those grown with sufficient levels of potassium (Figure 2A). This recognizable difference in gene expression between the plants during potassium deficient conditions and following resupply highlights the flexibility of biological pathways to adapt quickly as per changes in the environment. Through this study, we reveal adaptive responses of plants to fluctuations in potassium ion concentration. As previously reported, genes involved in various processes like primary and secondary metabolism, ion transport, signal transduction, and hormone signaling were linked to potassium deficiency [5], [44]–[47]. We have also identified differentially expressed genes related to carbohydrate metabolism, carboxyvinyl-carboxyphosphonate phosphorylmutase, glyoxalase, lactate/malate dehydrogenase, phosphoglycerate kinase, UDP-glucosyl transferase and many other enzymes and biological processes (Table 2). Many genes related to carbohydrate metabolism have also been reported to play important roles in response to phosphorus deficiency in rice roots and potassium deficiency [46], [48]. One of the genes identified in this study, UDP-glucosyl transferase, was also found to be influenced by iron toxicity in rice [49]. Similarly, alpha amylase transcript that shows the greatest magnitude of upregulation in response to potassium deficiency was also reported to be involved in starch hydrolysis [50].

Our microarray data suggests a number of proteins involved in diverse aspects of carbohydrate metabolism were differentially regulated and thereby indicates that potassium deficiency has widespread effects on carbohydrate metabolism. Carbohydrate metabolism directly impacts plant biomass and photosynthetic yield and, based on our findings, could be a significant component of the stress imposed by potassium deprivation.

Lipases are enzymes involved in the cleavage of lipids and are crucial components of lipid metabolism responsible for the mobilization of lipids during stress conditions. In this study, several lipases were identified that exhibited downregulation during potassium deprivation. In *Arabidopsis*, Armengaud et al. (2009) [5] reported the involvement of lipases and their similar differential expression in potassium deficiency treatments.

Based on earlier physiological analysis of growth symptoms under potassium deficiency, photosynthesis was identified as one of the major processes directly affected by potassium deficiency [51]–[53]. Photosynthesis serves as the major energy source of plants, and its efficiency is reduced under nutrient deficiencies [54]. The efflux of potassium ion from guard cells causes decreased stomatal conductance and thereby reduces rates of photosynthesis as a tradeoff for reduced water loss [51], [55]–[58]. In our differential gene expression analysis, we have identified several genes involved in photosynthesis. In particular, chlorophyll A–B binding protein and magnesium-protoporphyrin IX monomethyl ester cyclase were downregulated in KM conditions and strongly upregulated upon KR conditions. Similarly, several genes involved in jasmonic acid (JA), auxin, ethylene, and gibberellin signaling pathways are known to be directly influenced by potassium deficiency in plants [5], [37], [59].

With extensive research in the field of stress biology in plants, it is widely appreciated that many of these stresses are inter-related and produce similar secondary messenger molecules such as reactive oxygen species (ROS), calcium (Ca^2+^), and others [32], [54], [60]. Research in *Arabidopsis* pertaining to deprivation of multiple nutrients (N, S, P, K, Fe and others) suggests the activation of signaling pathways implicated in other abiotic stresses such as drought or toxic ion exposure [32], [61]. Presumably due to the interconnected nature of diverse plant stresses, plants grown under low potassium conditions have also been shown to be particularly susceptibile to drought and cold stress [9], [34], [36]. Moreover, many of the abiotic or biotic stresses affect metabolism and hence affect growth [62]. Different cellular processes like growth, photosynthesis, carbohydrate and lipid metabolism, osmotic homeostasis, protein synthesis are affected by both biotic and abiotic stresses [63]–[66].

Reactive oxygen species (ROS) are a byproduct of normal cell metabolism in plants. Accumulation of ROS is damaging to cells, however ROS also act as important signaling molecules. The balance between production and detoxification of ROS is tightly regulated by many proteins and enzymes [67]–[69]. ROS are generated as a signal of several biotic and abiotic stress conditions [68], [70]–[72]. ROS level were also elevated in response to deficiency of several nutrients (N, S, P, Fe), and ROS are a known signal of potassium starvation in roots [31], [32], [45], [73]. Under potassium-deficient conditions, many ROS related genes such as oxidoreductase and glutathione S-transferase (GST) are involved in detoxifying the ROS to maintain homeostasis of these molecules in the cytosol. We observed that transcripts encoding these enzymes were upregulated in response to potassium deprivation and subsequently downregulated following resupply of potassium.

Recently in the field of calcium signaling in plants, calcium has often been placed downstream of ROS [26], [31], [32], [40], [74], [75]. In previous reports in *Arabidopsis*, calcium-binding proteins were described to be differentially regulated in response to potassium starvation conditions [45]. Calcium sensors such as calmodulin (CaM), calcineurin B-like proteins (CBLs), CBL interacting protein kinases (CIPKs), and Ca^2+^-dependent protein kinases (CDPKs), were differentially expressed in potassium-deficient conditions [5]. In the rice root transcriptome, OsCIPK29 was found to be downregulated in response to potassium deficiency [37]. Three other CIPKs (OsCIPK7, OsCIPK8, and OsCIPK29) were found to be downregulated under potassium starvation. Their expression increased after resupply of potassium in this study, which suggests that these kinases might be acting as negative regulators of potassium nutrition and signaling in rice. One recently discovered, important aspect of potassium uptake and nutrition that has been elucidated in *Arabidopsis* is regulation of the high affinity K^+^ channel AKT1 by physical interactions with the calcium-binding proteins CBL1 and CBL9 and the Ser/Thr kinase CIPK23 in the root to regulate uptake of potassium [24], [25]. The downregulation of two PP2C protein phosphatases in the transcriptomic data suggests that these phosphatases might be acting as counteracting molecules in protein phosphorylation pathways that signal potassium-deficient conditions. Previously, it was reported that several members of the PP2C family are involved in regulating responses to multiple abiotic stresses as well as developmental processes in rice [76]. Some MAP kinases were also differentially regulated in potassium-deficient conditions, which suggest other signaling components are involved in the regulation of potassium starvation responses. MAPKs have also been implicated in both biotic and abiotic stress responses in addition to potassium starvation, development, cell proliferation, and hormone physiology [47], [61], [77], [78]. The involvement of phytohormones like JA and auxin in potassium-deficient conditions was shown earlier by identification of differentially regulated genes in the metabolism or signaling of these phytohormones [5], [37]. Surprisingly, we identified only a few candidate genes involved in JA and auxin metabolism and signaling during potassium-deficient conditions in rice seedling. It is quite possible that the altered expression of these signal transduction, stress and hormone-related genes might also play roles in enabling plants to adapt under low potassium stress conditions in rice.

Genome-wide analysis has led to the identification of a large family of genes encoding K^+^ transporters/channels in both *Arabidopsis* and rice [79]–[83]. Moreover, several detailed gene expression profiles by northern blots and RT-PCR analyses identified multiple K^+^ transporters that are differentially regulated under potassium starvation conditions [19], [61], [84]. However, in this transcriptomic analysis, we could not identify any K^+^ transporters with at least two fold change in gene expression, in contrast to the findings of Ma et al 2012 [37]. Similar to our findings, two previous reports on transcriptomic responses to potassium starvation in *Arabidopsis* did not identify many K^+^ transporters with significant changes in expression [5], [42]. Upon relaxing our analysis stringency to 1.5 fold with FDR, p<0.05, three transporters including HAK1 (LOC_Os04g32920) and KAT1 (LOC_Os01g11250) were identified. OsHAK1 is a member of the KUP/HAK/KT family of putative high-affinity K^+^ transporters that have been known to be involved in potassium uptake under low potassium conditions [37], [85]. KAT1 is a shaker-type K^+^ channel that is specifically expressed in guard cells and mediates potassium fluxes for turgor-dependent regulation of the stomatal aperture [86], [87]. As reported earlier, HKT class I transporters conduct Na^+^ uptake under KM condition in rice. In contrast, class II HKT transporters showed higher permeability for K^+^ ions. [88]–[93]. In transcriptomic analysis, the expression of sulfate transporters such as SULTR1;2 was found to be altered under potassium-deficient condition in *Arabidopsis* [5]. Approximately 30 DEG annotated as transporters that conduct molecules other than K^+^ were identified in this study, and these are believed to be involved in the transport of metals, lipids, sulphate, and other molecules. We identified representatives of ABC transporters, aquaporins, MATE efflux transpirters, and AAA-ATPase-type transporters. The differential expression of these transporters in potassium-deficient conditions might be related to the homeostasis of other molecules to maintain proper osmoticum and neutralize the charges across the membranes and in the cytosol [19], [32], [94].

The identification of fewer K^+^ transporters led to an argument for the possible existence of post-translational regulatory mechanisms of K^+^ transporters that direct uptake and distribution of potassium from the root to shoot. In fact, this mode of regulation has been recently discovered in *Arabidopsis*, where a high affinity K^+^ channel was regulated by a calcium-mediated phosphorylation-dephosphorylation network in roots [24], [25], [95]. However, no such phosphorylation-dephosphorylation signaling network has been identifed yet in rice. We speculate that similar post-translational modes of regulation are likely present in rice for regulation of K^+^ transporters during low potassium conditons.

In the present transcriptomic analysis, genes for a large number of transcription factors were differentially regulated in KM condition and their expression returned to baseline levels upon resupply of potassium. The maximum effect was shown by members of the zinc-finger transcription factor family, followed by MYB family transcription factors (Table 2). Zinc-finger family and basic helix-loop-helix (BHLH038, BHLH039, BHLH100, and BHLH101) transcription factors were previously reported to be involved in responses to iron deficiency in *Arabidopsis* [96]. Multiple reports in plants suggest that MYB family transcription factors regulate a wide range of processes in plants, including biotic and abiotic stress responses, plant growth and development, and secondary metabolite production [97]–[101]. In addition to potassium and iron, this family is also involved in phosphate starvation signaling in both vascular plants and unicellular algae [102].

Regulatory *cis* elements present in promoter regions are one of the salient aspects of gene regulation and often provide a mechanistic explanation for coexpression of a group of coexpressed genes. Several common *cis*-regulatory elements have been identified in multiple genes regulated similarly at transcriptional level [103]. Upon analysis of *cis*-regulatory elements in some of the highly differentially expressed genes in the present study, we found that they contained previously identifed drought responsive, sugar responsive, and light responsive regulatory *cis*-elements including ABRELATERD1, ACGTABOX, GT1CONSENSUS, GATABOX and others [104]–[107]. Interestingly, an earlier study concluded that ABREs functions Ca^2+^-responsive *cis*-elements in response to calcium transients induced by biotic and abiotic stresses [108]–[110]. Also the expressions of many potassium transporters and channels (KCO) have been shown to be activated by cytosolic Ca^2+^ [111]. The TAAAGSTKST1 element was found in promoter of the StKST1 gene encoding a K^+^ channel, and this gene is a known target site for trans-acting StDof1 protein controlling guard cell-specific gene expression [112]. Thus, the presence of these *cis*-regulatory elements in the promoter sequences of genes involved in nutrient stress might be associated with several abiotic and biotic stresses that lead to changes in the expression of signal transduction components and might also regulate signaling pathway crosstalk to enable plants to acclimatize to a wide range of conditions. Despite the presence of several common *cis*-regulatory elements in the promoters of highly differentially expressed genes under potassium deprivation, we could not identify a motif or *cis*-element specific for potassium responsiveness.

The comparative transcriptome analysis of rice and *Arabidopsis* (Figure S1, S2) suggests a common regulatory mechanism for genes whose expression is highly dependent on potassium availability. Such genes are implicated in a variety of cellular processes including metabolism, molecular transport, transcriptional regulation and signal transduction. We believe the concerted interplay of this diverse suite of genes coordinates plant adaptation to potassium starvation.

Cold, drought, and high salinity are common stress conditions that adversely affect plant growth and crop productivity. Due to abiotic stresses, the plant metabolism and homeostasis is also affetced. In order to achieve adjustment of metabolic pathways, different signaling networks also worked together [113]. Plant adaptation to environmental stresses is dependent upon the activation of various cascades of molecular networks involved in stress perception, signal transduction, and the expression of specific stress-related genes and metabolites [114]. Genes found in potassium deprivation condition were also involved in plant stress management. Carboxyvinyl-carboxyphosphonate phosphorylmutase showed 62 fold downregulation in potassium deficient condition, was also downregulated in different stress treatment. Also potassium responsive genes such as heparanase, UDP glucoronosyl, phosphoglycerate kinase and beta-amylase were also directly involved in plant stress management [115], [116], [117], [118]. Various reports revealed that genes involved in signal transduction like CML14, BRI I, MAPK were also responsive to several environmental stresses [119], [120], [121].

## Conclusions

In the present study, comprehensive gene expression analysis was performed using Affymetrix rice genome arrays during short-term potassium deficiency and resupply of potassium to deprived seedlings. A large number of genes were found to be differentially expressed. Taking into account the many potential consequences of the gene expression changes we observed, our study emphasizes the major metabolic adjustments associated with adaptation to the stress of nutrient deficiency. Potassium nutrition impacts a number of metabolic, signaling and physiological processes; together these processes coordinate plant adaptation to low potassium conditions.

**Figure 7:**
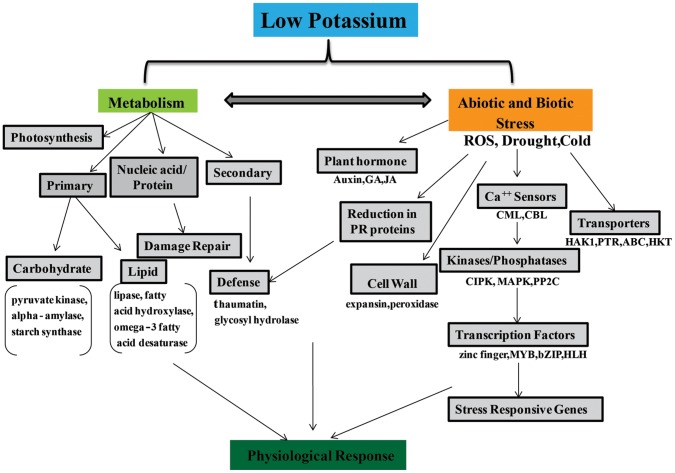
Model of plant responses and adaptation to potassium deficiency. Under low K^+^ conditions a large number of genes are affected. This condition alters plant metabolic as well as signaling responses. During K^+^ deprivation conditons, the initial reaction of plant is to sense the K^+^ deficiency followed by activation of several genes and gene networks, which might be working in tandem. As depected several genes encoding metabolic enzymes, protein involved in stress signaling, transporters, transcription factors could be the final target to bring about a physiological response such as tolerance or adaptation to low-K^+^ condition. Putative factors of K^+^ deficiency and adaptive responses are shown in boxes. Genes of related components are written below the boxes.

The overlap of low potassium responses with responses to biotic and abiotic stresses attests to the complex physiological changes triggered by nutrient starvation conditions. Under low potassium conditions, a large array of genes are activated, starting from sensing of nutrient deficiency in the soil to the transducing of this signal by activation of a plethora of signaling networks to, ultimately, the regulation of downstream proteins including transporters, enzymes, and transcription factors. We expect that the transcriptional changes in several diverse physiological and metabolic pathways are critical for adaptive responses to plant potassium deprivation conditon. Besides transcriptional regulation, post-translational modulation could certainly constitute another important mode of regulation in response to low potassium conditions. Despite the complexity of responses to potassium deficiency, we have made a hypothetical model to summarize the results of this study (Figure 7). As described in (Figure 7), low potassium influences metabolic processes and impacts genes related to different stress responses. Although, both processes are interconnected by various signaling pathways, the differential expression of individual components might affect the net signaling output of these highly interrelated processes. The changes in expression patterns of these components might govern the acclimation of the plant to such conditions. Further verification of these differentially expressed genes *in planta* by genetic and biochemical approaches will provide insights into the mechanistic regulation of these gene (s) during low potassium stress conditions, which ultimately can be translated to develop plants that can grow on soil with relatively low levels of potassium without compromising yield and productivity.

## Materials and Methods

### Plant Material and Growth Conditions

Seeds of indica rice (*Oryza sativa* subsp. *indica* cv. IR64) were surface sterilized and grown hydroponically in culture solution prepared as described [122]. The solution contained 1.14 mM NH_4_NO_3_, 0.258 mM NaH_2_PO_4_, 0.409 mM K_2_SO_4_, 0.798 mM CaCl_2_, 1.31 mM MgSO_4_, 0.0075 mM MnCl_2_, 0.06 µM (NH_4_)_6_Mo_7_O_24_, 15 µM H_3_BO_3_, 8 µM MnCl_2_, 0.12 µM CuSO_4_, 0.12 µM ZnSO_4_, 28 µM FeCl_3_, 61 µM citric acid, pH 4.7. Plants were grown in the rice growth room under the condition of 16 h light/8 h dark (28°C) photoperiod with 70% humidity. After 5 days of normal growth, one half of the rice seedlings were transferred to nutrient media with normal concentration of potassium (KP, 0.409 mM K_2_SO_4_) and other one half to medium without potassium (KM, 0 mM). After 5 days, potassium (0.409 mM K_2_SO_4_) was resupplied to the plants grown in KM medium for 6 h. The nutrient medium was changed at the interval of every 2 days. The seedlings were then harvested and immediately frozen in liquid nitrogen and stored at −80°C until total RNA isolation.

### Measurement of Total Ion Content

Seedlings treated with the above mentioned conditions of K^+^ concentration were collected and dried in oven at 80 degree for 24–48 h. To 100 mg of the dried tissue, 1 ml of spectroscopic grade concentrated HNO_3_ was added for the solubilisation. Samples were then incubated at room temperature (RT) for 2 days to ensure complete solubilisation of the tissue. The solubilized tissue was digested in the digester at 80° celcius temperature to vaporize almost 95% of the acid. The completely digested tissue was then transferred to a fresh micro-centrifuge tube and the volume was adjusted to a total of 1 ml using triple distilled milliQ water. The content was centrifuged at 10,000 g for 5 min and the supernatant was filtered using a 0.2 micron filter (Mdi, India). Three biological replicates were used for the statistical analysis. The concentrations of Na^+^ and K^+^ ions in seedling tissues were measured using a single-channel flame photometer (Digital Flame Analyzer model 2655-00; Cole-Parmer). In each analytical batch, reagent blanks and spiked samples were included in the acid digestion to assess the accuracy of the chemical analysis. The recovery of spike was 94–95% (n = 6). 100 µM and 40 µM solutions of NaCl and KCl were used as reference standards during analysis. Three biological replicates were analysed for each treatment.

### Measurement of Fresh and Dry Weight

A total of 15 rice seedlings were grown in normal (KP) and potassium deficient (KM) condition followed by weighing (fresh weight) separately and dried at 80°C for 48 h, and then again weighed (dry weight). DW/FW ratio was calculated and t - test (*P<0.05, **P<0.01) was used to analyze the statistical significance. Three biological replicates were used for fresh and dry weight measurement.

### RNA Extraction and Microarray Experiments

Total RNA was isolated from whole seedlings, except seeds, using the TRIzol method (Invitrogen Inc., USA), and RNA was further purified by Nucleospin RNA clean-up columns (Macherey-Nagel, Germany). The total RNA samples were quantified using nanodrop (ND-1000 Spectrophotometer (NanoDrop Technologies, USA ) and then analysed on denaturing MOPS agarose gel (2%) as well as by using Agilent Bioanalyser (Agilent Technologies, CA) for determining integrity and quality of RNA. After verifying the quality, 500 nanograms of RNA with 260∶280 ratios of 1.9–2.0 and 260∶230 ratios more than 2.0 was used for cDNA synthesis. Affymetrix GeneChip® Rice Genome Arrays representing 57,381 probe sets have been used to study the transcriptome profiles of potassium responsive genes in potassium deficient stress in rice. Labeling and hybridizations were carried out according to Affymetrix manual for one-cycle target labeling (Affymetrix, Santa Clara, CA). Hybridization was performed in a GeneChip® Hybridization oven 640 for 16 hours at 45°C and 60 rpm. GeneChips were washed using the fluidics protocol EukGE_WS2V5_450 in Affymetrix fluidic station model 450 and the chips were scanned using the Gene- Chip® Scanner 3000 (Affymetrix, Santa Clara, CA). Three biological replicates were used for each treatment.

### Microarray Data Analysis

The CEL files created by genechip operating software (GCOS) were imported into array assist (version 5.0) software (Stratagene, La Jolla, CA) for further analysis. GCRMA algorithm and log transformation were used for the normalization of microarray data. Student’s t-test was performed on log transformed value with the Benjamini-Hochberg correction to identify the differentially expressed genes. Benjamini-Hochberg correction ensured that transcripts showing significant changes were only analysed. Genes with fold change ≥2 and p<0.05 were considered to be significantly expressed. Oligonucleotide sequences of the probes were then mapped to Osa1 Rice Genome Annotation Project release 6 (RGAP: http://rice.plantbiology.msu.edu/) to annotate the differentially expressed genes. For hierarchical clustering, Euclidean distance metric and Wards linkage rule was used. Additional K-means clustering was carried out to identify the expression patterns of all differentially expressed genes in potassium-deficient conditions.

### Real-time PCR Analysis for the Validation of Microarray Data

Nineteen significantly differentially expressed genes under potassium deficiency were selected for quantitative real time PCR. The same RNA samples that were used for microarrays were also used for real time PCR. For all the selected genes primers were designed mainly from 3′ end of the gene using PRIMER EXPRESS version 2.0 (PE Applied Biosystems, USA) with default parameters. The primer sequences are listed in Table S4. Specificity of the primers was checked using BLAST tool of NCBI. First strand cDNA was prepared from 2 µg of DNase treated total RNA in 50 µl of reaction volume using high-capacity cDNA Archive kit (Applied Biosystems, USA). One µl of the first strand cDNA reaction was used for quantitative real time PCR. KAPA SYBR FAST Master Mix (KAPABIOSYSTEMS, USA) was used to determine the expression level for the genes in ABI Prism 7000 Sequence detection System (Applied Biosystems, USA). Three biological and three technical replicates were taken for each treatment, and standard deviation and standard error were calculated. As an endogenous control, *Actin* was used for the normalization of Ct value obtained and the relative expression values were calculated by ΔΔCt method.

## Supporting Information

Figure S1 **Comparison of functional category between rice and** ***Arabidopsis*** **in responses to K^+^ deficiency.**
(TIF)

Figure S2 **Comparison of functional category of rice seedling in responses to different nutrient deficiency.**
(TIF)

Figure S3 **Relation of potassium responsive DEGs with abiotic stresses.** (A) Genes related to metabolism category (B) Genes related to signal transduction category (C) Genes related to stress category.
(TIF)

Table S1 **722 genes showing fold change ≥2 in response to K^+^ starvation (P<0.05).**
(XLSX)

Table S2 **1876 genes showing fold change ≥2 in response to K^+^ resupply (P<0.05).**
(XLSX)

Table S3 **307 differentially expressed genes common in K^+^ starvation and K^+^ resupply.**
(XLSX)

Table S4 **List of primers used for real time PCR expression analysis.**
(XLSX)

Table S5 **Full category of 722 differentially expressed genes.**
(XLSX)

Table S6 **Genes showing fold change ≥1.5 in response to K^+^ starvation (P<0.05).**
(XLSX)

Table S7 **Common** ***cis*** **-regulatory elements found in highly expressive genes of** ***Arabidopsis*** **and rice in potassium deficient condition.**
(XLSX)

## References

[1] Clarkson DT, Hanson JB (1980) The mineral nutrition of higher plants. Annu Rev Plant Physiol 31: 239–298.

[2] Maathuis FJM, Sanders D (1996a) Mechanisms of potassium absorption by higher plant roots. Physiol Plant 96: 158–68.

[3] Zimmermann S, Sentenac H (1999) Plant ion channels: from molecular structure stophysiological functions.Curr Opin Plant Biol. 2: 477–82. 10.1016/s1369-5266(99)00020-5 10607654

[4] Very AA, Sentenac H (2003) Molecular mechanisms and regulation of K^+^ transport in higher plants. Annu Rev Plant Biol 54: 575–603. 14503004 10.1146/annurev.arplant.54.031902.134831

[5] Armengaud P, Breitling R, Amtmann A (2004) The potassium-dependent transcriptome of *Arabidopsis* reveals a prominent role of jasmonic acid in nutrient signaling. Plant Physiol 136: 2556–2576. 15347784 10.1104/pp.104.046482 PMC523322

[6] Luan S, Lan W, Chul Lee S (2009) Potassium nutrition, sodium toxicity, and calcium signaling: connections through the CBL-CIPK network. Curr Opin Plant Biol 12: 339–346. 19501014 10.1016/j.pbi.2009.05.003

[7] Tokas I, Pandey A, Pandey GK (2013) Role of Calcium-Mediated CBL–CIPK Network in Plant Mineral Nutrition and Abiotic Stress. *In* Molecular Stress Physiology of Plants Rout and Das (eds.) Springer India doi ––––10.1007/978–81–322–0807–5_10.

[8] Kochian LV, Shaff JE, Lucas WJ (1989) High-affinity K^+^ uptake in maize roots – a lack of coupling with H^+^ efflux. Plant Physiol 91: 1202–11. 16667133 10.1104/pp.91.3.1202 PMC1062141

[9] Marschner JP, Rietbrock N (1995) Oxygen release kinetics in healthy subjects and diabetic patients. II: Effects of HbCO. Int J Clin Pharmacol Ther 33: 263–265. 7655764

[10] Wyn Jones RJ, Pollard A (1983) Proteins, enzymes and inorganic ions. In A Lauchli, A Pirson, eds, Encyclopedia of Plant Physiol. Springer, Berlin, 528–562.

[11] Sorger GJ, Giles NH (1965) Genetic control of nitrate reductase in Neurospora crassa. Genetics 52: 777–788. 5826322 10.1093/genetics/52.4.777 PMC1210939

[12] Sorger GJ, Evans HJ (1966) Effects of univalent cations on the properties of yeast NAD^+^ acetaldehyde dehydrogenase. Biochim Biophys Acta 118: 1–8. 5954059 10.1016/s0926-6593(66)80139-x

[13] Nitsos RE, Evans HJ (1966) Effects of univalent cations on the inductive formation of nitrate reductase. Plant Physiol 41: 1499–1504. 5956844 10.1104/pp.41.9.1499 PMC550561

[14] Peoples TR, Koch DW (1979) Role of potassium in carbon dioxide assimilation in Medicago sativa L. Plant Physiol. 63: 878–881. 10.1104/pp.63.5.878 PMC542937 16660830

[15] Amtmann A, Bohnert HJ, Bressan RA (2005) Abiotic stress and plant genome evolution. Search for new models. Plant Physiol 138: 127–130. 15888685 10.1104/pp.105.059972 PMC1104168

[16] Ahn SJ, Shin R, Schachtman DP (2004) Expression of KT/KUP genes in *Arabidopsis* and the role of root hairs in K^+^ uptake. Plant Physiol 134: 1135–1145. 14988478 10.1104/pp.103.034660 PMC389937

[17] Alcazar R, Marco F, Cuevas JC, Patron M, Ferrando A, et al (2006) Involvement of polyamines in plant response to abiotic stress. Biotechnol Lett 28: 1867–1876. 17028780 10.1007/s10529-006-9179-3

[18] Schachtman DP, Schroeder JI (1994) Structure and transport mechanism of a high-affinity potassium uptake transporter from higher plants. Nature 370: 655–658. 8065452 10.1038/370655a0

[19] Gierth M, Maser P (2007) Potassium transporters in plants–involvement in K^+^ acquisition, redistribution and homeostasis. FEBS Lett 581: 2348–2356. 17397836 10.1016/j.febslet.2007.03.035

[20] Grabov A (2007) Plant KT/KUP/HAK potassium transporters: single family - multiple functions. Ann Bot 99: 1035–1041. 17495982 10.1093/aob/mcm066 PMC3243584

[21] Yao X, Horie T, Xue S, Leung HY, Katsuhara M, et al (2011) Differential sodium and potassium transport selectivities of the rice OsHKT2;1 and OsHKT2;2 transporters in plant cells. Plant Physiol 152: 341–355. 10.1104/pp.109.145722 PMC2799368 19889878

[22] Maathuis FJ, Sanders D (1994) Mechanism of high-affinity potassium uptake in roots of *Arabidopsis thaliana* . Proc Natl Acad Sci U S A 91: 9272–9276. 7937754 10.1073/pnas.91.20.9272 PMC44794

[23] Pyo YJ, Gierth M, Schroeder JI, Cho MH (2010) High-affinity K^+^ transport in *Arabidopsis*: AtHAK5 and AKT1 are vital for seedling establishment and post germination growth under low-potassium conditions. Plant Physiol 153: 863–875. 20413648 10.1104/pp.110.154369 PMC2879780

[24] Xu J, Li HD, Chen LQ, Wang Y, Liu LL, et al (2006) A protein kinase, interacting with two calcineurin B-like proteins, regulates K^+^ transporter AKT1 in *Arabidopsis* . Cell 125: 1347–1360. 16814720 10.1016/j.cell.2006.06.011

[25] Li L, Kim BG, Cheong YH, Pandey GK, Luan S (2006) A Ca^2+^ signaling pathway regulates a K^+^ channel for low-K response in *Arabidopsis* . Proc Natl Acad Sci U S A 103: 12625–12630. 16895985 10.1073/pnas.0605129103 PMC1567929

[26] Kasuga M, Liu Q, Miura S, Yamaguchi-Shinozaki K, Shinozaki K (1999) Improving plant drought, salt, and freezing tolerance by gene transfer of a single stress-inducible transcription factor. Nat Biotechnol 17: 287–291. 10096298 10.1038/7036

[27] Chen W, Provart NJ, Glazebrook J, Katagiri F, Chang HS, et al (2002) Expression profile matrix of *Arabidopsis* transcription factor genes suggests their putative functions in response to environmental stresses. Plant Cell 14: 559–574. 11910004 10.1105/tpc.010410 PMC150579

[28] Singh K, Foley RC, Onate-Sanchez L (2002) Transcription factors in plant defense and stress responses. Curr Opin Plant Biol 5: 430–436. 12183182 10.1016/s1369-5266(02)00289-3

[29] Cherel I, Michard E, Platet N, Mouline K, Alcon C, et al (2002) Physical and functional interaction of the *Arabidopsis* K^+^ channel AKT2 and phosphatase AtPP2CA. Plant Cell 14: 1133–1146. 12034902 10.1105/tpc.000943 PMC150612

[30] Lee SC, Lan WZ, Kim BG, Li L, Cheong YH, et al (2007) A protein phosphorylation/dephosphorylation network regulates a plant potassium channel. Proc Natl Acad Sci U S A 104: 15959–15964. 17898163 10.1073/pnas.0707912104 PMC2000415

[31] Shin R, Schachtman DP (2004) Hydrogen peroxide mediates plant root cell response to nutrient deprivation. Proc Natl Acad Sci U S A 101: 8827–8832. 15173595 10.1073/pnas.0401707101 PMC423280

[32] Shin R, Berg RH, Schachtman DP (2005) Reactive oxygen species and root hairs in *Arabidopsis* root response to nitrogen, phosphorus and potassium deficiency. Plant Cell Physiol 46: 1350–1357. 15946982 10.1093/pcp/pci145

[33] Mittler R, Vanderauwera S, Gollery M, Van Breusegem F (2004) Reactive oxygen gene network of plants. Trends Plant Sci 9: 490–498. 15465684 10.1016/j.tplants.2004.08.009

[34] Imas M, Imas P (2007) Evaluation of potassium compared to other osmolytes in relation to osmotic adjustment and drought tolerance of chickpea under water deficit environments. J Plant Nutr 30: 517–535.

[35] Sutter JU, Sieben C, Hartel A, Eisenach C, Thiel G, et al (2007) Abscisic acid triggers the endocytosis of the *Arabidopsis* KAT1 K^+^ channel and its recycling to the plasma membrane. Curr Biol 17: 1396–1402. 17683934 10.1016/j.cub.2007.07.020

[36] Benlloch-Gonzalez M, Romera J, Cristescu S, Harren F, Fournier JM, et al (2010) K^+^ starvation inhibits water-stress-induced stomatal closure via ethylene synthesis in sunflower plants. J Exp Bot 61: 1139–1145. 20054030 10.1093/jxb/erp379

[37] Ma TL, Wu WH, Wang Y (2012) Transcriptome analysis of rice root responses to potassium deficiency. BMC Plant Biol 12: 161. 22963580 10.1186/1471-2229-12-161 PMC3489729

[38] Higo K, Ugawa Y, Iwamoto M, Higo H (1998) PLACE: a database of plant cis-acting regulatory DNA elements. Nucleic Acids Res 26: 358–359. 9399873 10.1093/nar/26.1.358 PMC147199

[39] Mohanty B, Krishnan SP, Swarup S, Bajic VB (2005) Detection and preliminary analysis of motifs in promoters of anaerobically induced genes of different plant species. Ann Bot 96: 669–681. 16027132 10.1093/aob/mci219 PMC4247034

[40] Pandey GK, Cheong YH, Kim BG, Grant JJ, Li L, et al (2007) CIPK9: a calcium sensor-interacting protein kinase required for low-potassium tolerance in *Arabidopsis* . Cell Res 17: 411–421. 17486125 10.1038/cr.2007.39

[41] Gierth M, Maser P (2007) Potassium transporters in plants-involvement in K^+^ acquisition, redistribution and homeostasis. FEBS Lett 581: 2348–2356. 17397836 10.1016/j.febslet.2007.03.035

[42] Maathuis FJ, Filatov V, Herzyk P, Krijger GC, Axelsen KB, et al (2003) Transcriptome analysis of root transporters reveals participation of multiple gene families in the response to cation stress. Plant J 35: 675–692. 12969422 10.1046/j.1365-313x.2003.01839.x

[43] Rodriguez-Navarro A, Rubio F (2006) High-affinity potassium and sodium transport systems in plants. J Exp Bot 57: 1149–1160. 16449373 10.1093/jxb/erj068

[44] Hermans C, Hammond JP, White PJ, Verbruggen N (2006) How do plants respond to nutrient shortage by biomass allocation? Trends Plant Sci 11: 610–617. 17092760 10.1016/j.tplants.2006.10.007

[45] Schachtman DP, Shin R (2007) Nutrient sensing and signaling: NPKS. Annu Rev Plant Biol 58: 47–69. 17067284 10.1146/annurev.arplant.58.032806.103750

[46] Armengaud P, Sulpice R, Miller AJ, Stitt M, Amtmann A, et al (2009) Multilevel analysis of primary metabolism provides new insights into the role of potassium nutrition for glycolysis and nitrogen assimilation in *Arabidopsis* roots. Plant Physiol 150: 772–785. 19346439 10.1104/pp.108.133629 PMC2689955

[47] Wang Y, Wu WH (2010) Plant sensing and signaling in response to K^+^-deficiency. Mol Plant 3: 280–287. 20339156 10.1093/mp/ssq006

[48] Wasaki J, Yonetani R, Kuroda S (2003b) Transcriptomic analysis of metabolic changes by phosphorus stress in rice plant roots. Plant Cell Environ 26, 1515–1523.

[49] Quinet M, Vromman D, Clippe A, Bertin P, Lequeux H, et al (2012) Combined transcriptomic and physiological approaches reveal strong differences between short- and long-term response of rice (*Oryza sativa*) to iron toxicity. Plant Cell Environ 35: 1837–1859. 22506799 10.1111/j.1365-3040.2012.02521.x

[50] van der Maarel MJ, van der Veen B, Uitdehaag JC, Leemhuis H, Dijkhuizen L (2002) Properties and applications of starch-converting enzymes of the alpha-amylase family. J Biotechnol 94: 137–155. 11796168 10.1016/s0168-1656(01)00407-2

[51] Terry N, Ulrich A (1973) Effects of potassium deficiency on the photosynthesis and respiration of leaves of sugar beet under conditions of low sodium supply. Plant Physiol 51: 1099–1101. 16658474 10.1104/pp.51.6.1099 PMC366413

[52] Zhao DL, Oosterhuis DM, Bednarz CW (2001a) Influences of potassium deficiency on photosynthesis, chlorophyll content and chloroplast ultrastructure of cotton plants. Photosyntetica 39: 103–109.

[53] Basile B, Reidel EJ, Weinbaum SA, DeJong TM (2003) Leaf potassium concentration, CO_2_ exchange and light interception in almond trees (Prunus dulcis (Mill) D.A. Webb). Sci Hortic 98: 185–194.

[54] Wang N, Hua H, Eneji AE, Li Z, Duan L, et al (2012) Genotypic variations in photosynthetic and physiological adjustment to potassium deficiency in cotton (*Gossypium hirsutum*). J Photochem Photobiol B 110: 1–8. 22387141 10.1016/j.jphotobiol.2012.02.002

[55] Peaslee DE, Moss DN (1968) Stomatal conductivities in K^+^ deficient leaves of maize (*Zea mays L*.), Crop Sci. 8: 427–430.

[56] Graham RD, Ulrich A (1972) Potassium deficiency-induced changes in stomatal behavior, leaf water potentials, and root system permeability in *Beta rulgaris L*. Plant Physiol. 49: 105–109. 10.1104/pp.49.2.105 PMC365909 16657905

[57] Kanai S, Moghaieb RE, El-Shemy HA, Panigrahi R, Mohapatra PK, et al (2011) Potassium deficiency affects water status and photosynthetic rate of the vegetative sink in green house tomato prior to its effects on source activity. Plant Sci 180: 368–374. 21421382 10.1016/j.plantsci.2010.10.011

[58] Liu CH, Chao YY, Kao CH (2012) Abscisic acid is an inducer of hydrogen peroxide production in leaves of rice seedlings grown under potassium deficiency. Bot Stud 53: 229–237.

[59] Guardia MD, Belloch M (1980) Effects of potassium and gibberellic acid on stem growth of whole sunflower plants. Physiol Plant 49: 443–448.

[60] Karuppanapandian T, Moon JC, Kim C, Manoharan K, Kim W (2011) Reactive oxygen species in plants: their generation, signal transduction, and scavenging mechanisms. Aust J Crop Sci 5: 709–725.

[61] Wang YH, Garvin DF, Kochian LV (2002) Rapid induction of regulatory and transporter genes in response to phosphorus, potassium, and iron deficiencies in tomato roots. Evidence for cross talk and root/rhizosphere-mediated signals. Plant Physiol 130: 1361–1370. 12428001 10.1104/pp.008854 PMC166655

[62] Krasensky J, Jonak C (2012) Drought, salt, and temperature stress-induced metabolic rearrangements and regulatory networks. J Exp Bot 63: 1593–1608. 22291134 10.1093/jxb/err460 PMC4359903

[63] Dhindsa RS, Cleland RE (1975) Water stress and protein synthesis: I. Differential inhibition of protein synthesis. Plant Physiol 55: 778–781. 16659166 10.1104/pp.55.4.778 PMC541705

[64] Munns R (2002) Comparative physiology of salt and water stress. Plant Cell Environ 25: 239–250. 11841667 10.1046/j.0016-8025.2001.00808.x

[65] Beck EH, Fettig S, Knake C, Hartig K, Bhattarai T (2007) Specific and unspecific responses of plants to cold and drought stress. J Biosci 32: 501–510. 17536169 10.1007/s12038-007-0049-5

[66] Chaves MM, Flexas J, Pinheiro C (2009) Photosynthesis under drought and salt stress: regulation mechanisms from whole plant to cell. Ann Bot 103: 551–560. 18662937 10.1093/aob/mcn125 PMC2707345

[67] Sagi M, Fluhr R (2006) Production of reactive oxygen species by plant NADPH oxidases. Plant Physiol 141: 336–340. 16760484 10.1104/pp.106.078089 PMC1475462

[68] Vellosillo T, Vicente J, Kulasekaran S, Hamberg M, Castresana C (2010) Emerging complexity in reactive oxygen species production and signaling during the response of plants to pathogens. Plant Physiol 154: 444–448. 20921160 10.1104/pp.110.161273 PMC2948990

[69] Sharma P, Jha AB, Dubey RS, Pessarakli M (2012) Reactive oxygen species, oxidative damage, and antioxidative defense mechanism in plants under stressful conditions. J Bot doi:10.1155/2012/217037.

[70] Apel K, Hirt H (2004) Reactive oxygen species: metabolism, oxidative stress, and signal transduction. Annu Rev Plant Biol 55: 373–399. 15377225 10.1146/annurev.arplant.55.031903.141701

[71] Hernandez I, Alegre L, Munne-Bosch S (2004) Drought-induced changes in flavonoids and other low molecular weight antioxidants in *Cistus clusii* grown under Mediterranean field conditions. Tree Physiol 24: 1303–1311. 15339740 10.1093/treephys/24.11.1303

[72] Bhattacharjee S (2005) Reactive oxygen species and oxidative burst: Roles in stress, senescence and signal transduction in plants Currnt Scienc. 89: 7–10.

[73] Jung JY, Shin R, Schachtman DP (2009) Ethylene mediates response and tolerance to potassium deprivation in *Arabidopsis* . Plant Cell 21: 607–621. 19190240 10.1105/tpc.108.063099 PMC2660615

[74] Pandey GK, Grant JJ, Cheong YH, Kim BG, Li le G, et al (2008) Calcineurin-B-like protein CBL9 interacts with target kinase CIPK3 in the regulation of ABA response in seed germination. Mol Plant 1: 238–248. 19825536 10.1093/mp/ssn003

[75] Lan WZ, Lee SC, Che YF, Jiang YQ, Luan S (2011) Mechanistic analysis of AKT1 regulation by the CBL-CIPK-PP2CA interactions. Mol Plant 4: 527–536. 21596690 10.1093/mp/ssr031

[76] Singh A, Giri J, Kapoor S, Tyagi AK, Pandey GK (2010) Protein phosphatase complement in rice: genome-wide identification and transcriptional analysis under abiotic stress conditions and reproductive development. BMC Genomics 11: 435. 20637108 10.1186/1471-2164-11-435 PMC3091634

[77] Nakagami H, Pitzschke A, Hirt H (2005) Emerging MAP kinase pathways in plant stress signaling. Trends Plant Sci 10: 339–346. 15953753 10.1016/j.tplants.2005.05.009

[78] Armengaud P, Breitling R, Amtmann A (2010) Coronatine-insensitive 1 (COI1) mediates transcriptional responses of *Arabidopsis* thaliana to external potassium supply. Mol Plant 3: 390–405. 20339157 10.1093/mp/ssq012 PMC2845782

[79] Maser P, Thomine S, Schroeder JI, Ward JM, Hirschi K, et al (2001) Phylogenetic relationships within cation transporter families of *Arabidopsis* . Plant Physiol 126: 1646–1667. 11500563 10.1104/pp.126.4.1646 PMC117164

[80] Amrutha RN, Sekhar PN, Varshney RK, Kishor P (2007) Genome-wide analysis and identification of genes related to potassium transporter families in rice (*Oryza sativa*). Plant Sci 172: 708–721.

[81] Gupta M, Qiu X, Wang L, Xie W, Zhang C, et al (2008) KT/HAK/KUP potassium transporters gene family and their whole-life cycle expression profile in rice (*Oryza sativa*). Mol Genet Genomics 280: 437–452. 18810495 10.1007/s00438-008-0377-7

[82] Grabov A (2007) Plant KT/KUP/HAK potassium transporters: single family- multiple functions. Ann Bot 99: 1035–1041. 17495982 10.1093/aob/mcm066 PMC3243584

[83] Zhang Z, Zhang J, Chen Y, Li R, Wang H, et al (2012) Genome-wide analysis and identification of HAK potassium transporter gene family in maize (*Zea mays L*.). Mol Biol Rep 39: 8465–8473. 22711305 10.1007/s11033-012-1700-2

[84] Wang TB, Gassmann W, Rubio F, Schroeder JI, Glass AD (1998) Rapid Up-regulation of HKT1, a high-affinity potassium transporter gene, in roots of barley and wheat following withdrawal of potassium. Plant Physiol 118: 651–659. 9765551 10.1104/pp.118.2.651 PMC34841

[85] Banuelos MA, Garciadeblas B, Cubero B, Rodriguez-Navarro A (2002) Inventory and functional characterization of the HAK potassium transporters of rice. Plant Physiol 130: 784–795. 12376644 10.1104/pp.007781 PMC166606

[86] Nakamura RL, McKendree WL Jr, Hirsch RE, Sedbrook JC, Gaber RF, et al (1995) Expression of an *Arabidopsis* potassium channel gene in guard cells. Plant Physiol 109: 371–374. 7480337 10.1104/pp.109.2.371 PMC157599

[87] Ashley MK, Grant M, Grabov A (2006) Plant responses to potassium deficiencies: a role for potassium transport proteins. J Exp Bot 57: 425–436. 16364949 10.1093/jxb/erj034

[88] Rubio F, Gassmann W, Schroeder JI (1996) Response: high-affinity potassium uptake in plants. Science 273: 978–979. 17839588 10.1126/science.273.5277.978

[89] Liu W, Schachtman DP, Zhang W (2000) Partial deletion of a loop region in the high affinity K^+^ transporter HKT1 changes ionic permeability leading to increased salt tolerance. J Biol Chem 275: 27924–27932. 10821831 10.1074/jbc.M002056200

[90] Horie T, Yoshida K, Nakayama H, Yamada K, Oiki S, et al (2001) Two types of HKT transporters with different properties of Na^+^ and K^+^ transport in *Oryza sativa* . Plant J 27: 129–138. 11489190 10.1046/j.1365-313x.2001.01077.x

[91] Ren ZH, Gao JP, Li LG, Cai XL, Huang W, et al (2005) A rice quantitative trait locus for salt tolerance encodes a sodium transporter. Nat Genet 37: 1141–1146. 16155566 10.1038/ng1643

[92] Corratge C, Zimmermann S, Lambilliotte R, Plassard C, Marmeisse R, et al (2007) Molecular and functional characterization of a Na^+^-K^+^ transporter from the Trk family in the ectomycorrhizal fungus *Hebeloma cylindrosporum* . J Biol Chem 282: 26057–26066. 17626012 10.1074/jbc.M611613200

[93] Jabnoune M, Espeout S, Mieulet D, Fizames C, Verdeil JL, et al (2009) Diversity in expression patterns and functional properties in the rice HKT transporter family. Plant Physiol 150: 1955–1971. 19482918 10.1104/pp.109.138008 PMC2719131

[94] Walker DJ, Leigh RA, Miller AJ (1996) Potassium homeostasis in vacuolate plant cells. Proc Natl Acad Sci U S A 93: 10510–10514. 11607707 10.1073/pnas.93.19.10510 PMC38416

[95] Lan WZ, Wang W, Wang SM, Li LG, Buchanan BB, et al (2010) A rice high-affinity potassium transporter (HKT) conceals a calcium-permeable cation channel. Proc Natl Acad Sci U S A 107: 7089–7094. 20351263 10.1073/pnas.1000698107 PMC2872450

[96] Wang HY, Klatte M, Jakoby M, Baumlein H, Weisshaar B, et al (2007) Iron deficiency-mediated stress regulation of four subgroup Ib BHLH genes in *Arabidopsis thaliana* . Planta 226: 897–908. 17516080 10.1007/s00425-007-0535-x

[97] Stracke R, Werber M, Weisshaar B (2001) The R2R3-MYB gene family in *Arabidopsis thaliana* . Curr Opin Plant Biol 4: 447–456. 11597504 10.1016/s1369-5266(00)00199-0

[98] Cominelli E, Galbiati M, Vavasseur A, Conti L, Sala T, et al (2005) A guard-cell-specific MYB transcription factor regulates stomatal movements and plant drought tolerance. Curr Biol 15: 1196–1200. 16005291 10.1016/j.cub.2005.05.048

[99] Seo PJ, Xiang F, Qiao M, Park JY, Lee YN, et al (2009) The MYB96 transcription factor mediates abscisic acid signaling during drought stress response in *Arabidopsis* . Plant Physiol 151: 275–289. 19625633 10.1104/pp.109.144220 PMC2735973

[100] Dubos C, Stracke R, Grotewold E, Weisshaar B, Martin C, et al (2010) MYB transcription factors in *Arabidopsis* . Trends Plant Sci 15: 573–581. 20674465 10.1016/j.tplants.2010.06.005

[101] Seo PJ, Park CM (2010) MYB96-mediated abscisic acid signals induce pathogen resistance response by promoting salicylic acid biosynthesis in *Arabidopsis* . New Phytol 186: 471–483. 20149112 10.1111/j.1469-8137.2010.03183.x

[102] Rubio V, Linhares F, Solano R, Martin AC, Iglesias J, et al (2001) A conserved MYB transcription factor involved in phosphate starvation signaling both in vascular plants and in unicellular algae. Genes Dev 15: 2122–2133. 11511543 10.1101/gad.204401 PMC312755

[103] Dare AP, Schaffer RJ, Lin-Wang K, Allan AC, Hellens RP (2008) Identification of a cis-regulatory element by transient analysis of co-ordinately regulated genes. Plant Methods 4: 17. 18601751 10.1186/1746-4811-4-17 PMC2491621

[104] Gilmartin PM, Chua NH (1990) Spacing between GT-1 binding sites within a light-responsive element is critical for transcriptional activity. Plant Cell 2: 447–455. 2152170 10.1105/tpc.2.5.447 PMC159901

[105] Villain P, Mache R, Zhou DX (1996) The mechanism of GT element-mediated cell type-specific transcriptional control. J Biol Chem 271: 32593–32598. 8955086 10.1074/jbc.271.51.32593

[106] Toyofuku K, Umemura T, Yamaguchi J (1998) Promoter elements required for sugar-repression of the RAmy3D gene for alpha-amylase in rice. FEBS Lett 428: 275–280. 9654148 10.1016/s0014-5793(98)00518-3

[107] Simpson SD, Nakashima K, Narusaka Y, Seki M, Shinozaki K, et al (2003) Two different novel cis-acting elements of erd1, a clpA homologous *Arabidopsis* gene function in induction by dehydration stress and dark-induced senescence. Plant J 33: 259–270. 12535340 10.1046/j.1365-313x.2003.01624.x

[108] Kaplan B, Davydov O, Knight H, Galon Y, Knight MR, et al (2006) Rapid transcriptome changes induced by cytosolic Ca^2+^ transients reveal ABRE-related sequences as Ca^2+^-responsive cis elements in *Arabidopsis* . Plant Cell 18: 2733–2748. 16980540 10.1105/tpc.106.042713 PMC1626612

[109] Finkler A, Kaplan B, Fromm H (2007) Ca^2+^ responsive cis-elements in plants. Plant Signal Behav 2: 17–19. 19704800 10.4161/psb.2.1.3611 PMC2633890

[110] Whalley HJ, Sargeant AW, Steele JF, Lacoere T, Lamb R, et al (2011) Transcriptomic analysis reveals calcium regulation of specific promoter motifs in *Arabidopsis* . Plant Cell 23: 4079–4095. 22086087 10.1105/tpc.111.090480 PMC3246331

[111] Czempinski K, Zimmermann S, Ehrhardt T, Muller-Rober B (1997) New structure and function in plant K^+^ channels: KCO1, an outward rectifier with a steep Ca^2+^ dependency. EMBO J 16: 2565–2575. 9184204 10.1093/emboj/16.10.2565 PMC1169868

[112] Zimmermann S, Hartje S, Ehrhardt T, Plesch G, Mueller-Roeber B (2001) The K^+^ channel SKT1 is co-expressed with KST1 in potato guard cells–both channels can co-assemble via their conserved KT domains. Plant J 28: 517–527. 11849592 10.1046/j.1365-313x.2001.01177.x

[113] Mittler R (2006) Abiotic stress, the field environment and stress combination. Trends Plant Sci 11: 15–19. 16359910 10.1016/j.tplants.2005.11.002

[114] Huang CH, Chiou SH (2011) Proteomic analysis of upregulated proteins in *Helicobacter pylori* under oxidative stress induced by hydrogen peroxide. Kaohsiung J Med Sci 27: 544–553. 22208537 10.1016/j.kjms.2011.06.019 PMC11916125

[115] Kaplan F, Kopka J, Haskell DW, Zhao W, Schiller KC, et al (2004) Exploring the temperature-stress metabolome of *Arabidopsis* . Plant Physiol 136: 4159–4168. 15557093 10.1104/pp.104.052142 PMC535846

[116] Gadjev I, Vanderauwera S, Gechev TS, Laloi C, Minkov IN, et al (2006) Transcriptomic footprints disclose specificity of reactive oxygen species signaling in *Arabidopsis* . Plant Physiol 141: 436–445. 16603662 10.1104/pp.106.078717 PMC1475436

[117] Castillejo MA, Maldonado MA, Ogueta S, Jorrín JV, et al (2008) Proteomic analysis of responses to drought stress in sunflower (*Helianthus annuus*) leaves by 2DE gel electrophoresis and mass spectrometry. The Open Proteom J 1: 59–71.

[118] Morimoto S, Tateishi N, Matsuda T, Tanaka H, Taura F, et al (1998) Novel hydrogen peroxide metabolism in suspension cells of *Scutellaria baicalensis Georgi* . J Biol Chem 273: 12606–12611. 9575222 10.1074/jbc.273.20.12606

[119] Bajguz A (2009) Brassinosteroid enhanced the level of abscisic acid in *Chlorella vulgaris* subjected to short-term heat stress. J Plant Physiol 166: 882–886. 19062130 10.1016/j.jplph.2008.10.004

[120] Reddy AS, Ali GS, Celesnik H, Day IS (2011) Coping with stresses: roles of calcium- and calcium/calmodulin-regulated gene expression. Plant Cell 23: 2010–2032. 21642548 10.1105/tpc.111.084988 PMC3159525

[121] Sinha AK, Jaggi M, Raghuram B, Tuteja N (2011) Mitogen-activated protein kinase signaling in plants under abiotic stress. Plant Signal Behav 6: 196–203. 21512321 10.4161/psb.6.2.14701 PMC3121978

[122] Yoshida S, Forno DA, Cook JH Gomez KA (1976) Laboratory Manual for Physiological Studies of Rice. 3rd ed. International Rice Research Institute, Los Banos, Philippines.

