# Supplementary figures and images for: Gene Expression Analysis of Rice Seedling under Potassium Deprivation Reveals Major Changes in Metabolism and Signaling Components
Source: PLoS One. 2013 Jul 29;8(7):e70321. doi: 10.1371/journal.pone.0070321 (PMC3726378; doi:10.1371/journal.pone.0070321)

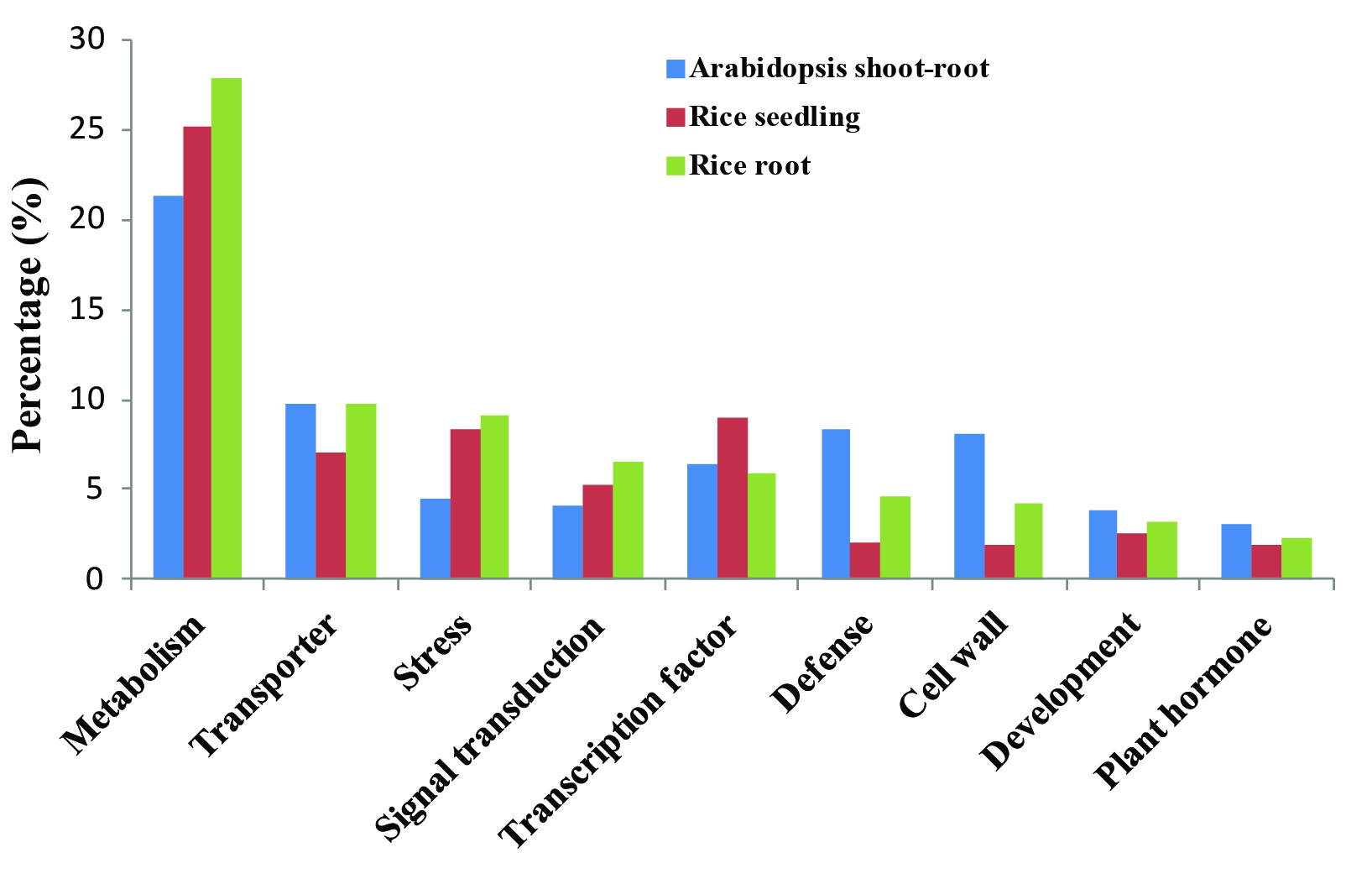

Supplement: Figure S1 — Comparison of functional category between rice and Arabidopsis in responses to K+ deficiency. (TIF) [file pone.0070321.s001.tif]

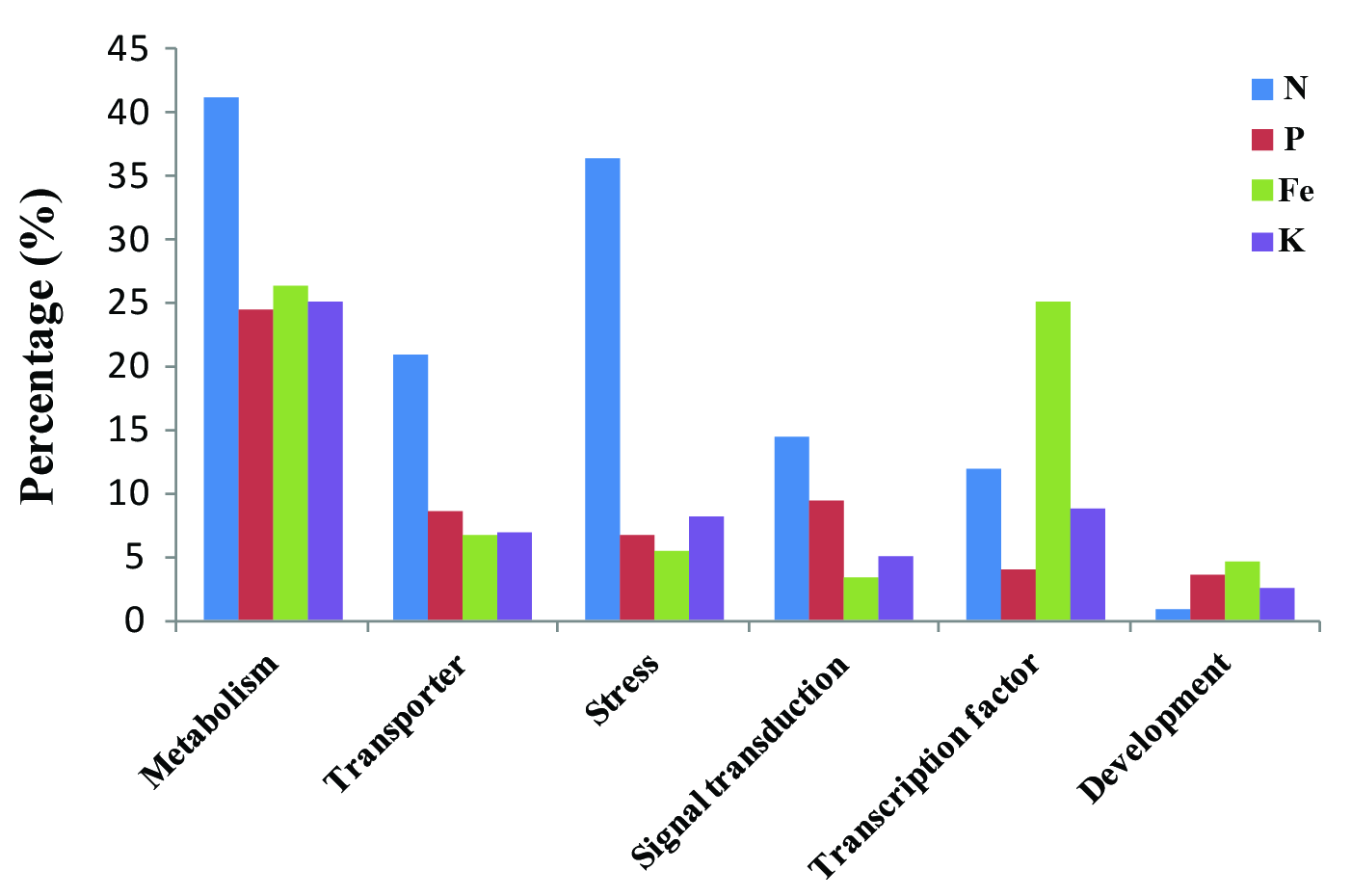

Supplement: Figure S2 — Comparison of functional category of rice seedling in responses to different nutrient deficiency. (TIF) [file pone.0070321.s002.tif]

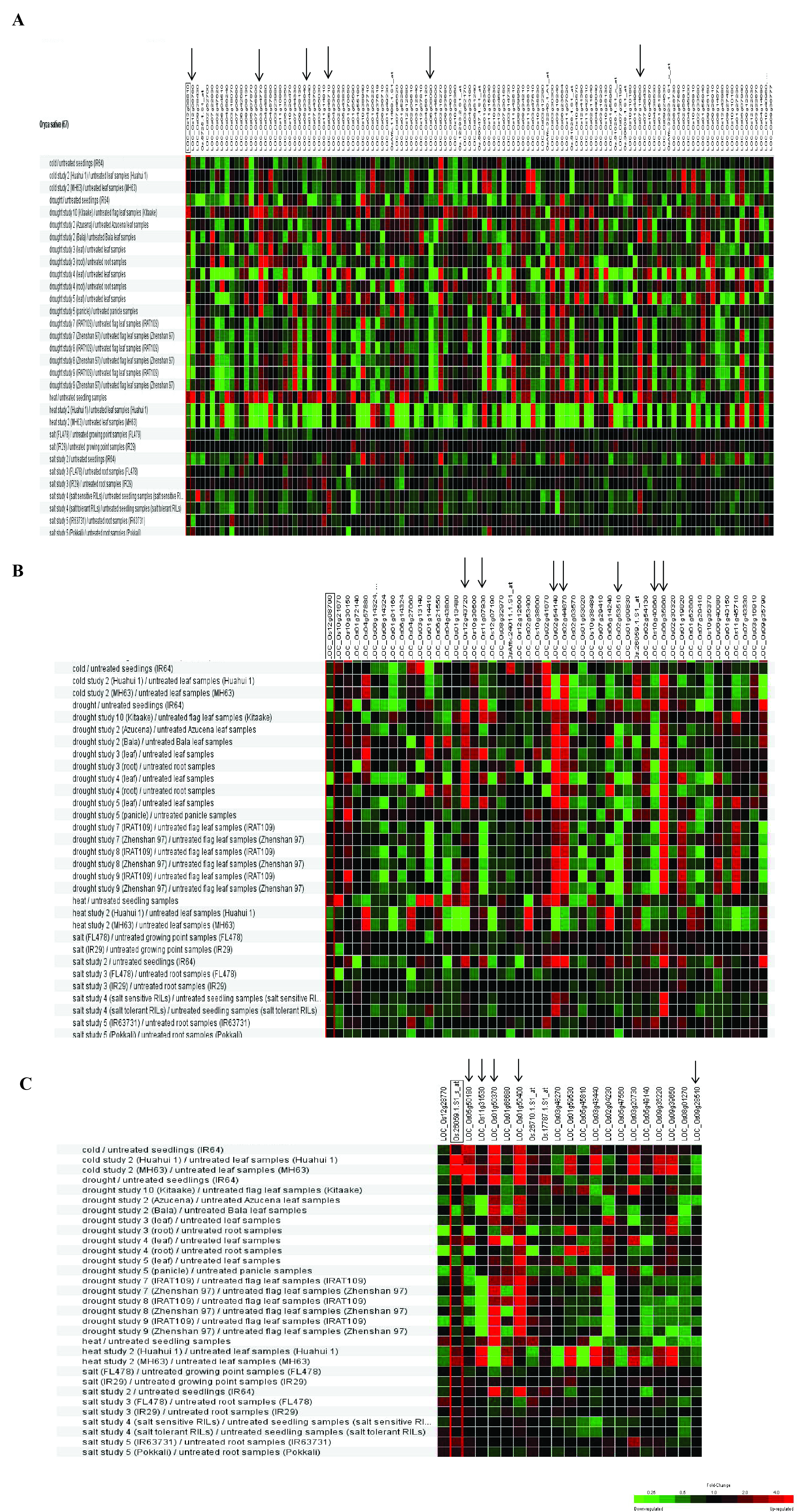

Supplement: Figure S3 — Relation of potassium responsive DEGs with abiotic stresses. (A) Genes related to metabolism category (B) Genes related to signal transduction category (C) Genes related to stress category. (TIF) [file pone.0070321.s003.tif]
